# Activation of eIF4E‐binding‐protein‐1 rescues mTORC1‐induced sarcopenia by expanding lysosomal degradation capacity

**DOI:** 10.1002/jcsm.13121

**Published:** 2022-11-17

**Authors:** Elisa M. Crombie, Seonyoung Kim, Stuart Adamson, Han Dong, Tzu‐Chiao Lu, Yiju Wu, Yajun Wu, Yotam Levy, Nolan Stimple, Wing Moon R. Lam, Hwee Weng D. Hey, Dominic J. Withers, Ao‐Lin Hsu, Boon Huat Bay, Julien Ochala, Shih‐Yin Tsai

**Affiliations:** ^1^ Department of Physiology, Yong Loo Lin School of Medicine National University of Singapore Singapore; ^2^ Buck Institute for Research on Aging Novato California USA; ^3^ Research Center for Healthy Aging China Medical University Taichung Taiwan; ^4^ Department of Anatomy, Yong Loo Lin School of Medicine National University of Singapore Singapore; ^5^ Centre of Human and Applied Physiological Sciences, School of Basic and Medical Biosciences, Faculty of Life Sciences & Medicine King's College London UK; ^6^ Department of Orthopedic Surgery, Yong Loo Lin School of Medicine National University of Singapore Singapore; ^7^ Metabolic Signalling Group Medical Research Council London Institute of Medical Sciences (LMS) London UK; ^8^ Institute of Clinical Sciences (ICS) Faculty of Medicine, Imperial College London London UK; ^9^ Institute of Biochemistry and Molecular Biology National Yang Ming Chiao Tung University Taipei Taiwan; ^10^ Department of Biomedical Sciences University of Copenhagen Copenhagen Denmark; ^11^ Healthy Longevity Translational Research Programme, Yong Loo Lin School of Medicine National University of Singapore Singapore

**Keywords:** mitochondrial dysfunction, mRNA translation, mTORC1, protein degradation, sarcopenia

## Abstract

**Background:**

Chronic mTORC1 activation in skeletal muscle is linked with age‐associated loss of muscle mass and strength, known as sarcopenia. Genetic activation of mTORC1 by conditionally ablating mTORC1 upstream inhibitor TSC1 in skeletal muscle accelerates sarcopenia development in adult mice. Conversely, genetic suppression of mTORC1 downstream effectors of protein synthesis delays sarcopenia in natural aging mice. mTORC1 promotes protein synthesis by activating ribosomal protein S6 kinases (S6Ks) and inhibiting eIF4E‐binding proteins (4EBPs). Whole‐body knockout of S6K1 or muscle‐specific over‐expression of a 4EBP1 mutant transgene (4EBP1mt), which is resistant to mTORC1‐mediated inhibition, ameliorates muscle loss with age and preserves muscle function by enhancing mitochondria activities, despite both transgenic mice showing retarded muscle growth at a young age. Why repression of mTORC1‐mediated protein synthesis can mitigate progressive muscle atrophy and dysfunction with age remains unclear.

**Methods:**

Mice with myofiber‐specific knockout of TSC1 (TSC1mKO), in which mTORC1 is hyperactivated in fully differentiated myofibers, were used as a mouse model of sarcopenia. To elucidate the role of mTORC1‐mediated protein synthesis in regulating muscle mass and physiology, we bred the 4EBP1mt transgene or *S6k1* floxed mice into the TSC1mKO mouse background to generate 4EBP1mt‐TSC1mKO or S6K1‐TSC1mKO mice, respectively. Functional and molecular analyses were performed to assess their role in sarcopenia development.

**Results:**

Here, we show that 4EBP1mt‐TSC1mKO, but not S6K1‐TSC1mKO, preserved muscle mass (36.7% increase compared with TSC1mKO, *P* < 0.001) and strength (36.8% increase compared with TSC1mKO, *P* < 0.01) at the level of control mice. Mechanistically, 4EBP1 activation suppressed aberrant protein synthesis (two‐fold reduction compared with TSC1mKO, *P* < 0.05) and restored autophagy flux without relieving mTORC1‐mediated inhibition of ULK1, an upstream activator of autophagosome initiation. We discovered a previously unidentified phenotype of lysosomal failure in TSC1mKO mouse muscle, in which the lysosomal defect was also conserved in the naturally aged mouse muscle, whereas 4EBP1 activation enhanced lysosomal protease activities to compensate for impaired autophagy induced by mTORC1 hyperactivity. Consequently, 4EBP1 activation relieved oxidative stress to prevent toxic aggregate accumulation (0.5‐fold reduction compared with TSC1mKO, *P* < 0.05) in muscle and restored mitochondrial homeostasis and function.

**Conclusions:**

We identify 4EBP1 as a communication hub coordinating protein synthesis and degradation to protect proteostasis, revealing therapeutic potential for activating lysosomal degradation to mitigate sarcopenia.

## Introduction

Sarcopenia is the age‐related loss of muscle quantity and quality alongside reduced muscle performance.^1^ Moreover, sarcopenia is a risk factor for frailty, falls and pathological conditions, including respiratory and neurodegenerative diseases, correlating with overall mortality.^2^ Muscle mass is regulated by a coordinated balance between anabolic (i.e., protein synthesis) and catabolic (i.e., protein breakdown) signalling, where a chronic increase in net muscle protein accretion or depletion results in muscle hypertrophy or atrophy, respectively. Intuitively, the solution to sarcopenia should be simple: induce muscle hypertrophy by stimulating muscle protein synthesis and/or lowering protein breakdown. Yet, it has been well established that elderly individuals exhibit a blunted protein synthetic response to anabolic stimuli known as ‘anabolic resistance’.^3^, ^4^ Therefore, elucidation of the molecular mechanisms involved in the regulation of muscle protein homeostasis is essential for effective therapeutic targeting of sarcopenia.

The basal rate of muscle protein synthesis does not decline during aging; instead, the sensitivity of muscle to anabolic stimuli is diminished.^5^ The mechanistic target of rapamycin (mTOR) is a Ser/Thr kinase that integrates anabolic stimuli, including growth factors and nutrients, to regulate cell proliferation and growth by promoting protein synthesis.^6^ mTOR is involved in two functionally distinct complexes known as mTORC1 and mTORC2. Whereas both complexes have been implicated in promoting protein synthesis,^7^ the role of mTORC1 is more well defined. Although it was initially hypothesized that constitutively increasing mTORC1 activity by knockout of its upstream inhibitor TSC1 in muscle would increase protein synthesis and result in muscle hypertrophy, a sarcopenic phenotype was observed.^8^, ^9^, ^10^ Consistent with the early‐onset sarcopenia developed in myofiber‐specific knockout of TSC1 (TSC1mKO) mice, multiple lines of evidence indicate chronic up‐regulation of mTORC1 signalling in the muscle of aged mice and humans.^10^, ^11^

The exact mechanisms underlying why muscle growth does not correspond with increased protein synthesis with age or in the TSC1mKO mouse model are not clear. The canonical targets of mTORC1 in the regulation of protein synthesis are S6 kinases (S6Ks) and eIF4E‐binding proteins (4EBPs).^12^ mTORC1 phosphorylates and activates S6Ks, which subsequently stimulates ribosomal biogenesis and the mRNA translation machinery. In parallel, mTORC1 activates cap‐dependent translation by phosphorylating and inhibiting 4EBPs, leading to its dissociation from eIF4E to initiate translation. Reducing protein synthesis through whole‐body knockout of S6K1 or myofiber‐specific activation of 4EBP1 leads to muscle atrophy at a young age. Yet, both mouse lines were shown to improve age‐related metabolic parameters in skeletal muscle, including preservation of insulin sensitivity, increased oxidative capacity and even maintenance of muscle mass with age.^13^, ^14^, ^15^, ^16^, ^17^

The present study aims to determine the contribution of S6K1 and 4EBP1 to mTORC1‐induced sarcopenia. Here, we found that myofiber‐specific activation of 4EBP1, but not inactivation of S6K1, restored muscle strength and preserved muscle mass in the TSC1mKO mouse background. Functionally, 4EBP1 activation not only reset the global protein synthesis rate but also unexpectedly expanded lysosomal degradation capacity, offsetting proteostasis and preventing the build‐up of toxic waste in the muscle of mice with chronic mTORC1 activation. Our data reveal a mechanistic coordination of protein synthesis and degradation via 4EBP1 in the regulation of muscle mass and function with age.

## Materials and methods

### Animals

The myofiber‐specific knockout and transgenic mice were generated by breeding conditional flox mice with Ckmm‐Cre transgenic mice. The puromycin incorporation assay to label newly synthesized protein was performed as previously described.^14^ Detailed mouse information is listed in *Table* *S1*. Detailed descriptions of the assessment of mouse and muscle physiology and metabolism are presented in the supporting information. Unless otherwise specified, mice were analysed at 12 months of age.

### Reagents/buffers and antibodies

The reagents/buffers and antibodies used for the study are listed in *Table* *S2*.

### Protein isolation and western blot

The snap‐frozen muscle was homogenized in ice‐cold radioimmunoprecipitation assay (RIPA) lysis buffer (0.1% sodium dodecyl sulfate [SDS]) with protease and phosphatase inhibitors, sonicated (Bioruptor Plus, Diagenode) for 5 s and centrifuged at 21 100 ×g for 30 min at 4°C. The supernatant after centrifugation was collected as the soluble fraction. The pellet was then further homogenized in lysis buffer (2% SDS), sonicated for 30 s for 10 cycles and centrifuged at 21 100 ×g for 30 min at 4°C, where supernatant was collected as the insoluble fraction. The western blotting was performed as described previously.^14^ Ponceau S staining was used for the quantification of total protein loading.

### Cap (m^7^GTP) pull‐down assay

Limb muscle from 4‐month‐old male mice was homogenized in cold buffer A with protease and phosphatase inhibitors, and the Cap (m^7^GTP) pull‐down assay was performed as previously described.^14^

### Cathepsin activity assay

Lysosomal proteinase (cathepsin) activity was determined in muscle acid lysate (MAL) as previously described.^18^

### Immunohistochemistry and immunofluorescence staining

All staining techniques described here were performed on 15‐μm‐thick muscle sections on poly‐d‐lysine‐coated slides. The full descriptions of the staining procedures are presented in the supporting information.

### Transmission electron microscopy

Transmission electron microscopy (TEM) was performed as described previously,^19^ with some application‐specific modifications. At the point of tissue harvest, muscle was immersed TEM fixed solution at 4°C for 4 h. Tissues were rinsed in 0.1‐M phosphate buffer (with 5% sucrose). Samples were post‐fixed in 1% osmium tetroxide in 0.1‐M phosphate buffer (pH 7.4) for 2 h at room temperature (RT) and dehydrated with an ascending ethanol series at RT. Tissue blocks were infiltrated in 100% acetone:resin (1:6) overnight with three changes of pure resin in a series of ascending temperature (40°C, 50°C and 55°C) for 1 h each. Samples were embedded in a pure araldite mixture at 60°C for 24 h. Cross‐sections of muscle were cut at 99‐nm thickness. Ultrathin sections were doubly stained in lead citrate and 3% uranyl acetate and viewed with a JEM‐1220 TEM (JEOL).

### RNA extraction and RT‐PCR analysis

Total RNA was extracted from 2‐month‐old male mouse limb muscles, and the analysis of transcription was performed as previously described.^14^ qPCR primer sequences are listed in *Table* *S3*.

### Polysome profiling

Limb muscles from 2‐month‐old male mice were used for polysome profiling. The description of polysome profiling is presented in the supporting information.

### RNA‐seq analysis

Total RNA (transcriptome) or polysome‐associated RNA (translatome) were sent to NovogeneAIT for RNA‐seq analysis using Illumina High Throughput Sequencing PE150. For the translatome analysis, *n* = 3 samples (pooled muscles from two mice were used per sample) were used per group. For the transcriptome analysis, *n* = 7 mice for control and *n* = 3 mice for TSC1mKO were used. A detailed description on bioinformatics analysis of RNA‐seq data can be found in the supporting information.

### Statistics

Unless otherwise stated, all results are expressed as mean ± SEM of independent animals. The significance of differences between multiple groups was evaluated by either one‐way or two‐way ANOVA followed by Tukey's post hoc pairwise comparison. All statistics were performed, and graphs were made using GraphPad Prism 9 software, with significance set at *P* < 0.05.

### Study approval

Animal studies were conducted at the National University of Singapore or Buck Institute for Research on Aging, following the Institutional Animal Care and Use Committee guidelines.

## Results

### Myofiber activation of 4EBP1 alleviated sarcopenia development in TSC1mKO mice

**Figure 1:**
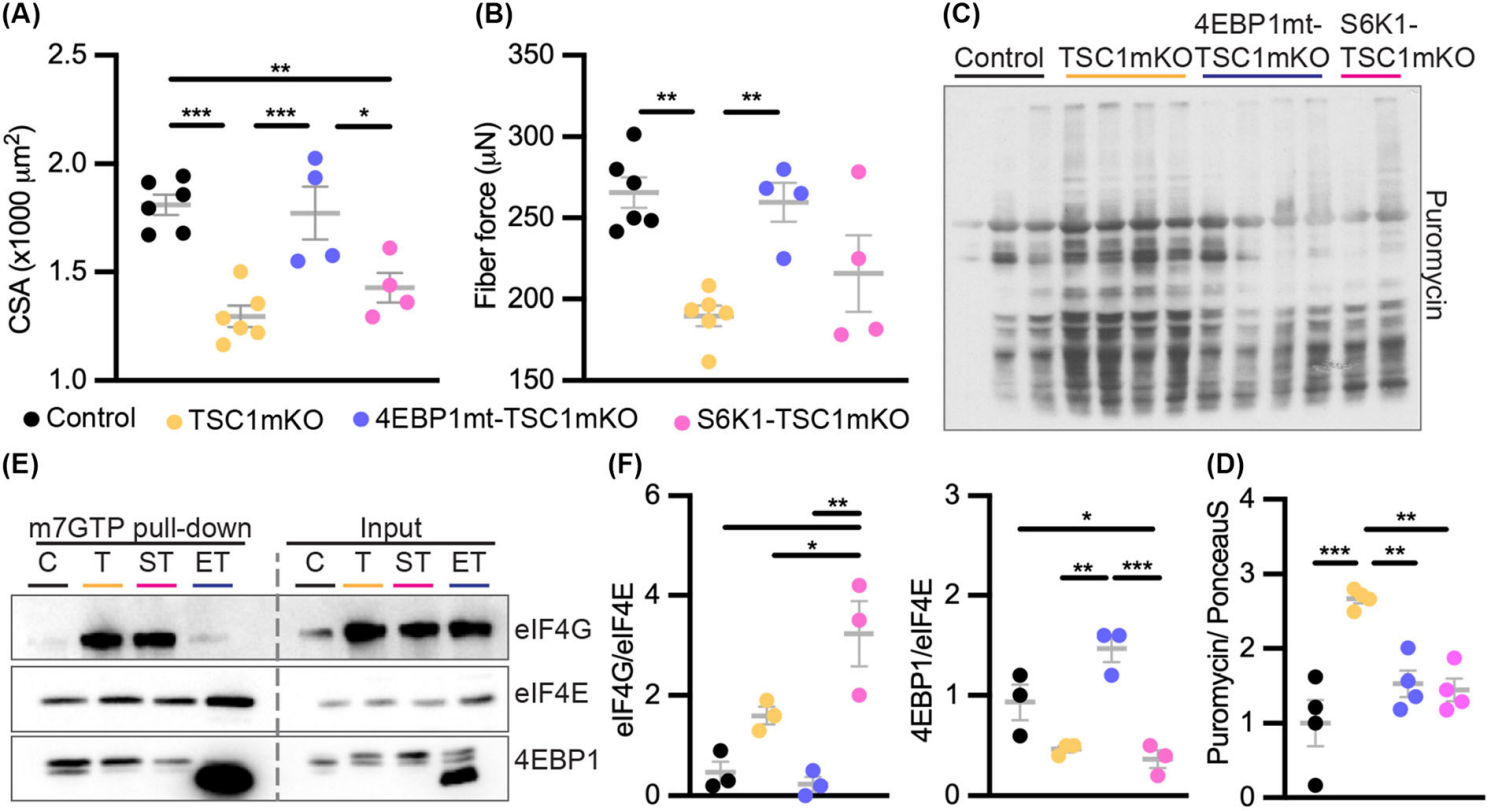
The sarcopenic features observed in myofiber‐specific knockout of TSC1 (TSC1mKO) mice were attenuated by eIF4E‐binding‐protein‐1 (4EBP1) activation. (A) Myofiber cross‐sectional area (CSA) and (B) myofiber force were measured from individual myofibers dissected from the tibialis anterior muscle of 12‐month‐old male mice (control *n* = 6, TSC1mKO *n* = 6, 4EBP1mt‐TSC1mKO *n* = 4, S6K1‐TSC1mKO *n* = 4). (C) Western blot analysis of puromycin incorporation in 4‐month‐old male mouse quadriceps muscle (QM). Ponceau S membrane staining was used as a loading control shown in *Figure* *S2D*. (D) The normalized quantification of puromycin incorporation immunoblotting from the blot in *Figures* *1C* and *S2E*. (E) 7‐Methylguanosine triphosphate (m^7^GTP) pull‐down assay on muscle lysates from 4‐month‐old male mice. Representative western blot is shown. Brief: C, control; T, TSC1mKO; ST, S6K1‐TSC1mKO; ET, 4EBP1mt‐TSC1mKO. (F) The quantification of the ratio of eIF4G and eIF4E (left) and the ratio of 4EBP1 and eIF4E (right) from m^7^GTP pull‐down assay from *Figures* *1E* and *S2F*. *n* = 3 mice per genotype. Data are shown as mean ± SEM and individual points correspond to one mouse. Statistical significance was determined by one‐way ANOVA with Tukey's multiple comparison test: *P < 0.05, **P < 0.01 and ***P < 0.001.

Dysregulated mTORC1‐S6K‐S6 signalling was reported in aged humans.^5^, ^10^ Consistently, myofiber‐restricted inactivation of TSC1 leads to sustained mTORC1 activity (TSC1mKO mice) and accelerates the development of sarcopenia in adult mice.^8^, ^9^, ^10^ By 12 months of age, TSC1mKO mice showed reduced myofiber size and force, measured by the force generated from the interaction of the actin–myosin in fast‐twitch tibialis anterior muscles (*Figure* *1* *A,B*). Muscle weakness in TSC1mKO mice was attributed to myofiber atrophy because a change in specific force (absolute force normalized to myofiber cross‐sectional area) was not detected (*Figure* *S2A*).

We previously demonstrated that over‐expression of a mutant 4EBP1 transgene (4EBP1mt), which is resistant to mTORC1‐mediated phosphorylation and inhibition in skeletal muscle, increased mitochondria biogenesis and autophagy activities and, in turn, delayed aging‐associated muscle pathologies.^14^ To gain further insight of 4EBP1 activation in mitigating pathological changing with muscle aging, the 4EBP1mt transgene was bred into the TSC1mKO mouse background to generate 4EBP1mt‐TSC1mKO mice. Additionally, we bred *S6k1* floxed mice into the TSC1mKO mouse background to generate S6K1‐TSC1mKO mice to examine the contribution of another downstream effector of mTORC1‐mediated regulation of protein synthesis to sarcopenia development (*Figure* *S2B,C*).

Because S6K1 and 4EBP1 are regulators of translation, we used puromycin, a structural analogue of aminoacyl‐tRNA, to label newly synthesis protein in vivo. Consistent with a previous report,^8^ global protein synthesis was up‐regulated (*Figure* *1* *C,D*) accompanied by increased phosphorylation of S6 and 4EBP1 (*Figure* *S2C*), prior to the development of histological myopathy in young TSC1mKO mice. Functionally, inactivation of S6K1 or activation of 4EBP1 lowered levels of global protein synthesis induced by chronic activation of mTORC1 (*Figure* *1* *C,D*). Further analyses showed that mTORC1‐mediated phosphorylation of S6K1 was significantly reduced in S6K1‐TSC1mKO mouse muscle, yet phosphorylation of ribosomal protein S6 was no different to that in TSC1mKO mice (*Figure* *S2B,C*). Downstream of mTORC1, both S6K1 and S6K2 can phosphorylate S6,^20^, ^21^ although S6K1 has been shown to regulate muscle growth and insulin sensitivity independent of S6K2 activity.^21^, ^22^ In the present study, all genetic mouse groups showed increased phosphorylation and thus activity of S6K2 (*Figure* *S2B*). Thus, although reduced global protein synthesis was observed in S6K1‐TSC1mKO mouse muscle, S6K2 activity may be sufficient to substitute S6K1 in phosphorylating S6, or there might be another kinase downstream of the mTORC1 pathway compensating for the loss of S6K1 in this mouse model.

During protein synthesis, the translation machinery recognizes a 7‐methylguanosine triphosphate (m^7^GTP) cap structure at the 5′ end of mRNA, which recruits eukaryotic translation initiation factors. mTORC1 phosphorylates 4EBP1 and blocks its binding to eIF4E, allowing eIF4E to associate with the eIF4G complex and initiate cap‐dependent translation. In young TSC1mKO mouse muscle, there was more eIF4G and less 4EBP1 associated with the eIF4E–m^7^GTP complex (*Figure* *1* *E,F*), indicating the increased formation of the cap‐dependent translation initiation complex. In 4EBP1mt‐TSC1mKO mouse muscle, 4EBP1mt protein sequestered eIF4E from binding with eIF4G, disrupting formation of the cap‐dependent translation initiation complex. Although eIF4E was increased in 4EBP1mt‐TSC1mKO mouse muscle (*Figure* *S2B,C*), the association of eIF4G and eIF4E–m^7^GTP complex was still reduced, assuring constitutive activity of 4EBP1 in repression cap‐dependent translation. Conversely, m^7^GTP–eIF4E–eIF4G complex formation was increased in S6K1‐TSC1mKO mouse muscle compared with control and even TSC1mKO mice, alongside partially up‐regulated 4EBP1 phosphorylation (*Figures* *1* *E,F* and *S2B*). These data indicate that feedback regulation of mTORC1 targets activities may exist and contribute to altered translational signalling.

Phenotypically, we found that only 4EBP1 activation, not S6K1 inactivation, restored myofiber size and force in the TSC1mKO background in adult mice (*Figure* *1* *A,B*). Moreover, increased curvature of the spine, potentially due to loss of back extensor strength, was prominent in adult TSC1mKO mice as previously reported^9^, ^10^ and was rescued by 4EBP1 activation (*Figure* *S2G,H*). Together, these data indicated that 4EBP1 activation alleviates sarcopenia in the TSC1mKO mouse model of sarcopenia, similar to that in naturally aged mice.^14^

### Myofiber activation of 4EBP1 relieved oxidative stress in TSC1mKO mouse muscle

**Figure 2:**
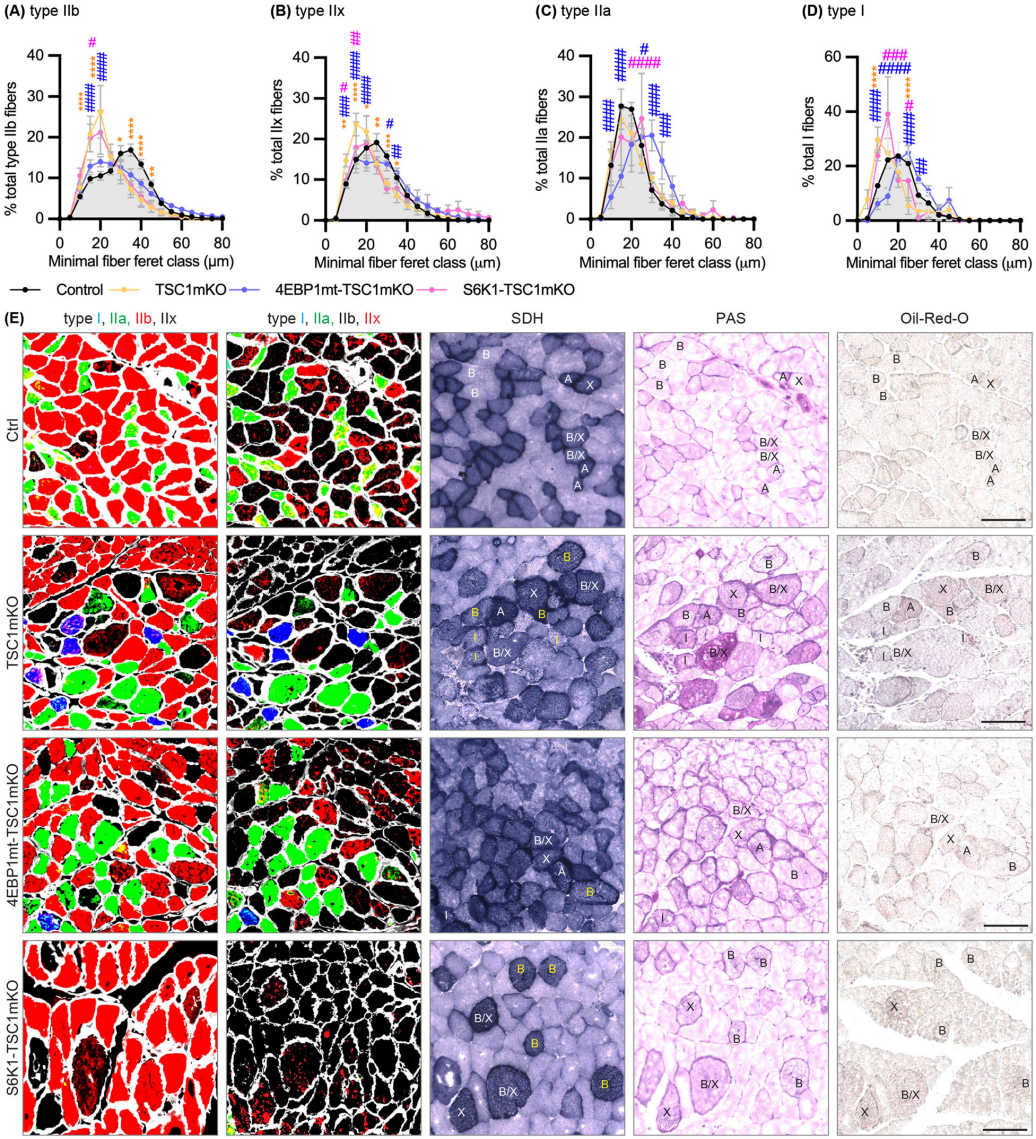
Myofiber activation of eIF4E‐binding‐protein‐1 (4EBP1) rescued type II myofiber atrophy and metabolic stress in myofiber‐specific knockout of TSC1 (TSC1mKO) mouse background. Quantification of immunofluorescence staining of (A) type IIb, (B) type IIx, (C) type IIa and (D) type I in cross‐sections of quadriceps muscle (QM) from 12‐month‐old male mice (control *n* = 4, TSC1mKO *n* = 4, 4EBP1mt‐TSC1mKO *n* = 4, S6K1‐TSC1mKO *n* = 3). Myofiber size is quantified by minimum Feret diameter, with ~2500–8500 myofibers analysed per mouse. Data are shown as mean ± SEM and statistical significance was determined by two‐way ANOVA with Tukey's multiple comparison test compared within the size class. *P < 0.05, **P < 0.01, ***P < 0.001 and ****P < 0.0001 indicate statistical significance of TSC1mKO versus control mice; ^#^ *P* < 0.05, ^##^ *P* < 0.01, ^###^ *P* < 0.001 and ^####^ *P* < 0.0001 indicate significance of 4EBP1mt‐TSC1mKO (blue) or S6K1‐TSC1mKO (pink) versus TSC1mKO mice. (E) Colocalization analysis of multiple stains on serial sections of 12‐month‐old male mouse gastrocnemius muscle (GM); scale bar, 100 μm. Myosin heavy chain (MyHC) staining was analysed by either combination of type IIa and type I with either type IIb (first column) or type IIx (second column). Succinate dehydrogenase (SDH) staining is used for assessment of mitochondrial activity (third column). Periodic acid‐Schiff (PAS) staining is used for assessment of glycogen content (fourth column) and Oil‐Red‐O staining is used for assessment of fatty acid deposit (fifth column). Corresponding muscle types are labelled in SDH, PAS and Oil‐Red‐O images for easy reference. Yellow font indicates abnormal type IIb myofibers with intense SDH staining.

Sarcopenia is characterized by type II fibre atrophy. In line with this phenotype, adult TSC1mKO mice showed specific atrophy of type IIb (fast‐twitch, glycolytic) fibres, with no major effect on type I (slow‐twitch, oxidative) fibres in the two biggest hindlimb muscles: quadriceps muscle (QM) and gastrocnemius muscle (GM) (*Figures* *2* *A–D* and *S3A–D*). Notably, TSC1mKO mice showed size variation in the type IIb population, where some fibres were atrophied and others showed compensatory hypertrophy (*Figure* *2* *E*). Haematoxylin and eosin (H&E) staining in TSC1mKO mouse muscle revealed basophilic small angular fibres and large rounded fibres containing inclusions or central nuclei (*Figure* *S3E*), indicative of pathological muscle degeneration and regeneration. There were no apparent effects of S6K1 knockout in the TSC1mKO background, whereas type IIb fibres in 4EBP1mt‐TSC1mKO mice were of similar size to control mice (*Figures* *2* *A* and *S3A*).

**Figure 3:**
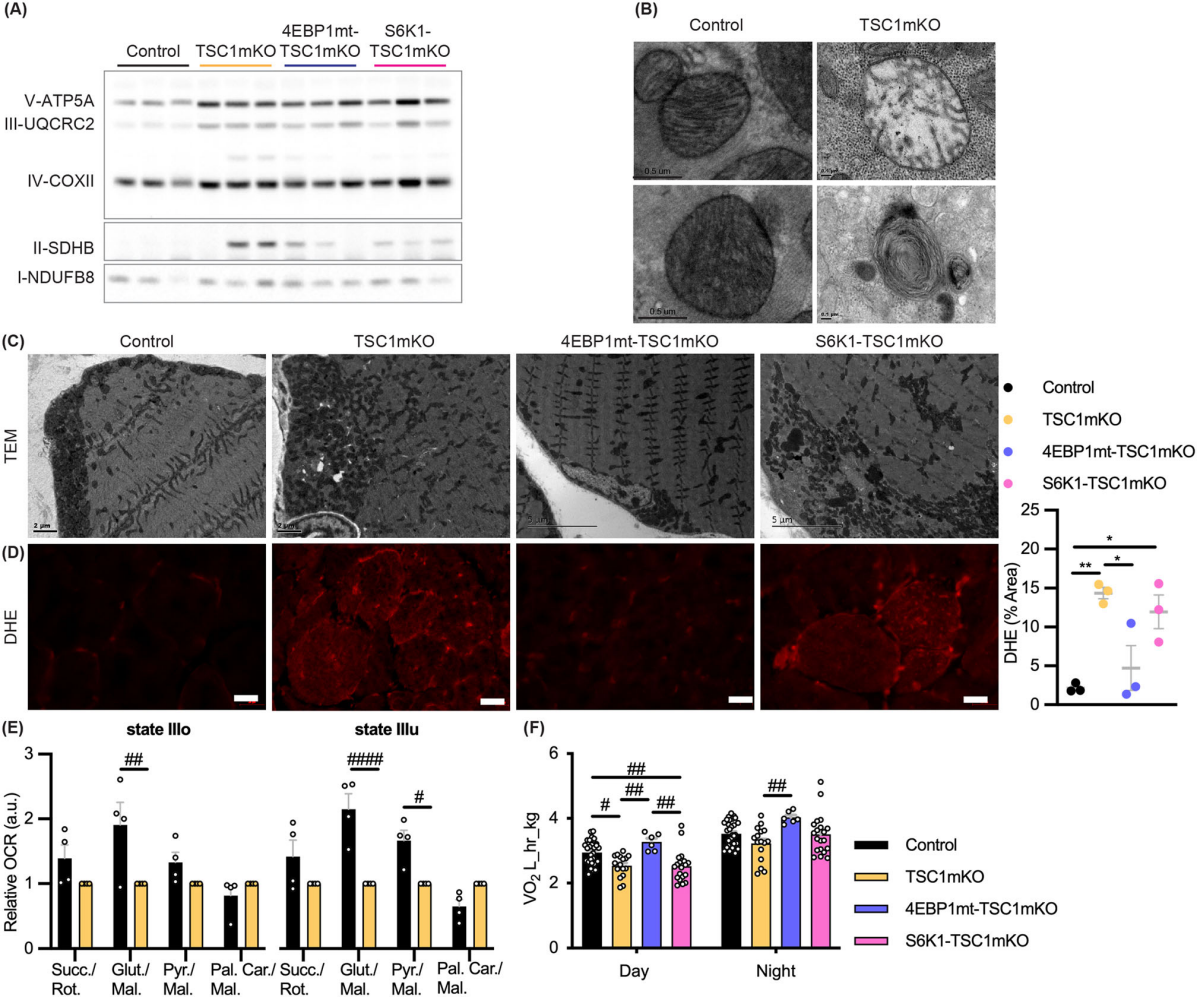
Myofiber activation of eIF4E‐binding‐protein‐1 (4EBP1) counteracted oxidative stress under conditions of chronic mTORC1 activity. (A) Immunoblotting for OXPHOS proteins from 12‐month‐old male mouse quadriceps muscle (QM), where each representative protein from each mitochondrial complex is indicated. Ponceau S membrane staining was used as a loading control shown in *Figure* *S3H* and the accompany blots with additional samples are presented in *Figure* *S3I*. *n* = 4 mice per genotype. The quantification of *Figures* *3A* and *S3I* is presented in *Table* *S4*. (B) Transmission electron microscopy (TEM) imaging of soleus muscle (SM) of 12‐month‐old male mice fasted for 18 h. Mitochondria with degenerated cristae (top) and concentric membrane structures (bottom) were found in myofiber‐specific knockout of TSC1 (TSC1mKO) mouse muscle. (C) TEM imaging of myofiber cross‐sections, shown for 12‐month‐old male mouse SM. Representative of three to four mice per genotype. (D) Dihydroethidium (DHE) staining of 12‐month‐old male mouse QM, shown by excitation at 594 nm (red); scale bar, 20 μm. Quantification of DHE staining of QM muscle from 12‐month‐old male mice is shown in the right (control *n* = 3, TSC1mKO *n* = 3, 4EBP1mt‐TSC1mKO *n* = 3, S6K1‐TSC1mKO *n* = 3). (E) Seahorse analysis of oxygen consumption rate (OCR) indicates the relative State IIIo and State IIIu (maximal uncoupler‐stimulated respiration) oxygen consumption for isolated mitochondria; normalized to OCR of each treatment for TSC1mKO mice as a plate control. (Brief: Succ./Rot., succinate with rotenone; Glut./Mal., glutamate with malate; Pyr./Mal., pyruvate with malate; Pal. Car./Mal., palmitoyl carnitine with malate.) Female (12‐month‐old) mouse muscle was used in this experiment (control *n* = 4, TSC1mKO *n* = 4). (F) Metabolic cage for analysis of oxygen consumption during day and night cycles from 12‐month‐old male mice (control *n* = 15, TSC1mKO *n* = 8, 4EBP1mt‐TSC1mKO *n* = 3, S6K1‐TSC1mKO *n* = 6). Data are shown as mean ± SEM and individual points correspond to one mouse in graphs. Statistical significance was determined by one‐way ANOVA with Tukey's multiple comparison test: *P < 0.05 and **P < 0.01; and by two‐way ANOVA across rows with Tukey's multiple comparison test: ^#^ *P* < 0.05, ^##^ *P* < 0.01 and ^####^ *P* < 0.0001.

Myosin heavy chain (MyHC) expression is often correlated with the metabolic properties of muscle. Succinate dehydrogenase (SDH) staining was used to determine the mitochondrial content of muscle, with heavy staining indicative of oxidative myofibers. In healthy mouse skeletal muscle, relative SDH staining is shown to be lowest in type IIb fibres, intermediate in type I fibres and highest in type IIa and type IIx fibres.^23^ Unexpectedly, we found that a portion of type IIb fibres strongly stained for SDH in TSC1mKO mice and S6K1‐TSC1mKO mice. Those heavily SDH‐stained type II fibres appeared either large and rounded or small and angular in shape and often strongly co‐stained with both periodic acid‐Schiff (PAS) and Oil‐Red‐O, indicating the accumulation of respiratory substrates glycogen and fatty acids, respectively (*Figures* *2* *E* and *S3F*). Consistent with SDH staining, increased mitochondrial proteins were detected, indicating that TSC1mKO mouse muscle contained more mitochondria than control mice (*Figure* *3* *A*). However, mitochondrial protein levels were not correlated with transcription (*Figure* *S3G*), suggesting that increased mitochondrial protein in adult TSC1mKO mice was due to either increased translation of mitochondrial genes or reduced mitochondrial degradation.

In healthy myofibers, mitochondria are organized into two functionally distinct subpopulations: subsarcolemmal mitochondria, localized at the myofiber periphery, and intermyofibrillar mitochondria, integrated into a uniform pattern surrounding myofibers to supply adenosine triphosphate (ATP) for contraction. In adult TSC1mKO mouse muscle, mitochondrial organization was disrupted, showing accumulation of subsarcolemmal mitochondria and clustering of intermyofibrillar mitochondria (*Figure* *3* *C*). Additionally, mitochondria with degenerated membranes or cristae were identified in TSC1mKO mouse muscle (*Figure* *3* *B*). Such ultrastructural abnormalities in mitochondrial organization were observed in patients with mitochondrial myopathy and correlated with impaired mitochondrial function.^24^ Mitochondrial dysfunction results in reactive oxygen species generation, an indication of oxidative stress, which was elevated in TSC1mKO mouse muscle (*Figure* *3* *D*), indicating an accumulation of defective mitochondria as previously reported.^10^ Functional analysis of mitochondria by Seahorse XF analysis showed that State IIIo (adenosine diphosphate [ADP] stimulated) and State IIIu (maximal respiration induced by carbonyl cyanide 4‐(trifluoromethoxy)phenylhydrazone [FCCP]) respiration rates were reduced in TSC1mKO mice (*Figure* *3* *E*), confirming the notion that defective mitochondria were accumulated. The mitochondrial defect was further validated at the whole‐body level by metabolic cage, which showed a significantly lower oxygen consumption rate in TSC1mKO mice (*Figure* *3* *F*).

Overall, the mitochondrial defects identified in TSC1mKO mice were rescued in 4EBP1mt‐TSC1mKO but not S6K1‐TSC1mKO mice. Enlarged rounded SDH/PAS/Oil‐Red‐O positive type II fibres were rarely observed (*Figure* *2* *E*) and mitochondrial organization was normalized (*Figure* *3* *C*) in 4EBP1mt‐TSC1mKO mouse muscle. Furthermore, oxidative stress was ameliorated (*Figure* *3* *D*) and systemic energy expenditure was restored in 4EBP1mt‐TSC1mKO mouse (*Figure* *3* *F*). Together, these data indicated that metabolic dysfunction in TSC1mKO mice resulted from the accumulation of damaged mitochondria that was restored by 4EBP1 activation.

**Figure 4:**
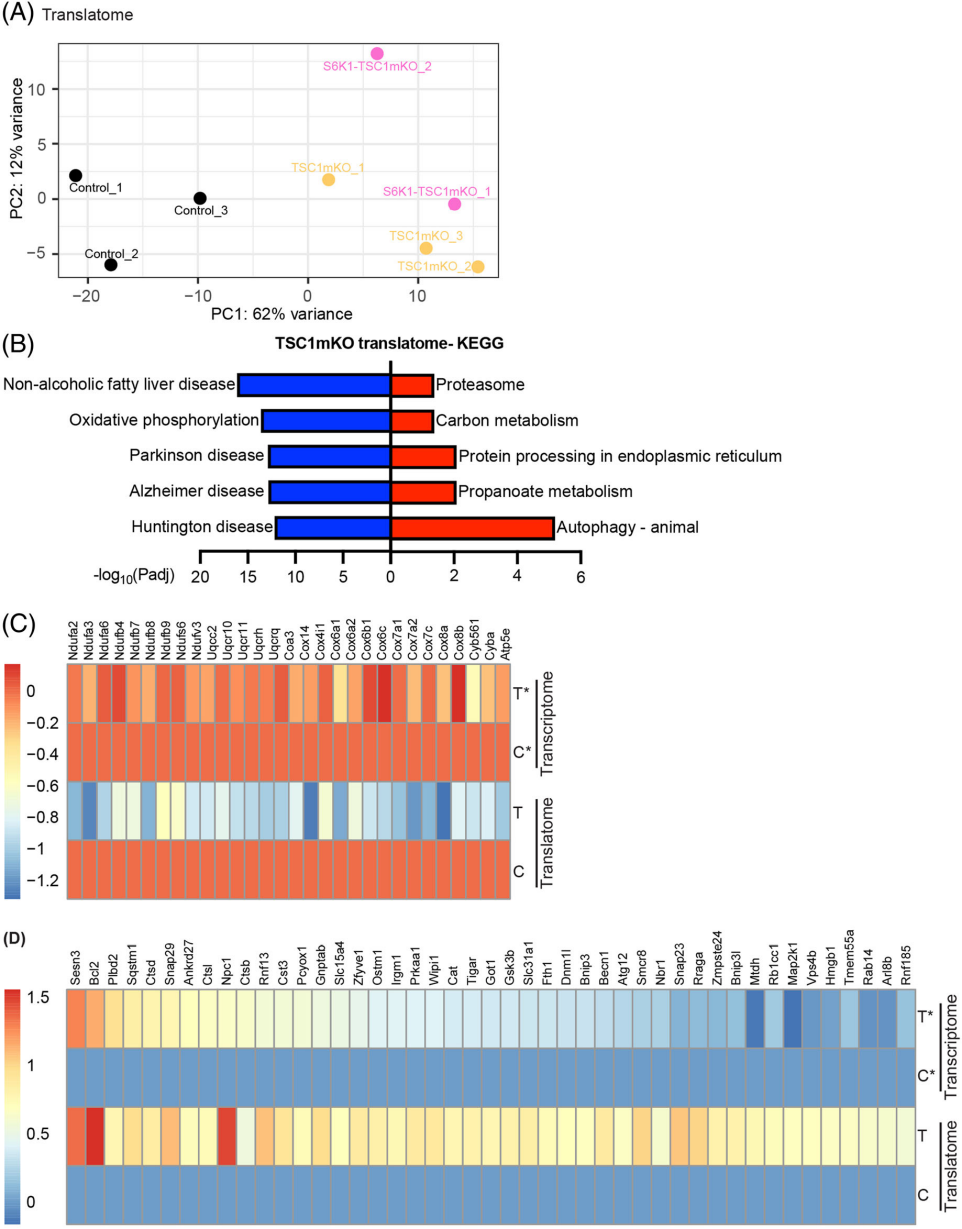
mTORC1‐driven transcriptome and translatome analysis in 2‐month‐old male mouse skeletal muscle. (A) Principal component analysis (PCA) of translatome data from RNA‐seq of polysome RNA from 2‐month‐old male mice. Each dot corresponds to one polysome sample where genotypes are indicated in colour. (B) Top‐five Kyoto Encyclopedia of Genes and Genomes (KEGG) gene ontology terms enriched (*P*adj < 0.001) for genes down‐regulated (labelled in blue) and up‐regulated (labelled in red) in myofiber‐specific knockout of TSC1 (TSC1mKO) translatome. (C) Heatmap of selected genes only down‐regulated in TSC1mKO translatome and are not changed in the transcriptome. (D) Heatmap of autophagic–lysosomal genes up‐regulated in TSC1mKO translatome and transcriptome. For the translatome analysis, *n* = 3 samples (*n* = 2 mice per sample) per group. For the transcriptome analysis, *n* = 7 mice were used for control and *n* = 3 mice for TSC1mKO groups.

Because mTORC1 has a central role in mRNA translation and activation of 4EBP1, a repressor of cap‐dependent translation, relieved the accumulation of damaged mitochondria, we first investigated whether translation was responsible for the increased mitochondria abundance in TSC1mKO mice. Because the translation rate significantly declines with age, we used younger mice (2 months of age) for this analysis in which the skeletal muscle is at a stage of exponential growth. Total RNA (transcriptome) and mRNA associated with polysomes (translatome) were extracted for RNA‐seq analysis (*Figures* *4* and *S4*). Due to the lower global translational rate in 4EBP1‐activated skeletal muscle, we could only analyse the translatome in control, TSC1mKO and S6K1‐TSC1mKO mouse muscles. In agreement with the physiology phenotypes reported above, principal component analysis indicated that chronic mTORC1 activation results in a distinct translational signature, where S6K1 inactivation had no major effect (*Figure* *4* *A*).

Translatome analysis identified that a group of genes that encode mitochondrial electron transport chain complexes were down‐regulated in TSC1mKO mouse muscle (*Figures* *4* *B,C* and *S4E*), suggesting that mitochondrial biogenesis was not increased, and that reduced mitochondrial degradation might be the cause of mitochondrial accumulation in TSC1mKO mice. The primary mechanism of mitochondrial degradation is mitophagy via the autophagy–lysosome system. Paradoxically, gene ontology enrichment analysis of the TSC1mKO translatome identified up‐regulation of genes involved in several protein degradation pathways, including the autophagic–lysosomal and the ubiquitin–proteasome systems (*Figures* *4* *B* and *S4E*). Moreover, a significant proportion of autophagic–lysosomal genes were also up‐regulated in the transcriptome of TSC1mKO mouse muscle (*Figure* *4* *E*). These findings suggested that mitochondrial and autophagic processes were highly perturbed due to chronic mTORC1 activation, which may drive the TSC1mKO phenotype. Because we did not observe a significant effect of S6K1 deletion on myopathy or a distinct molecular signature in the TSC1mKO mouse background, we focused on 4EBP1mt‐TSC1mKO mice for subsequent experiments.

### Myofiber activation of 4EBP1 reinstated autophagy–lysosome activities in TSC1mKO mouse muscle

**Figure 5:**
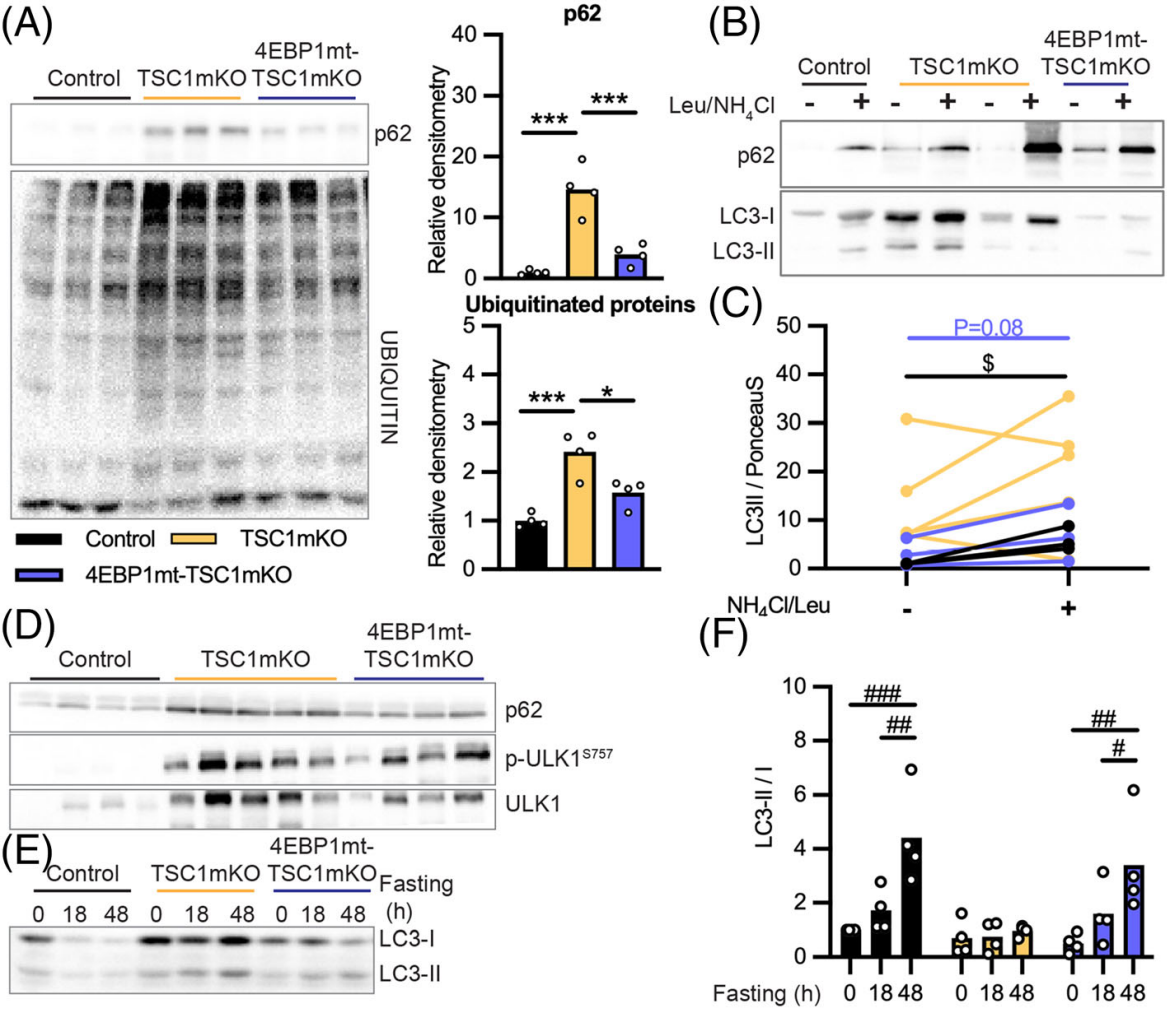
Myofiber activation of eIF4E‐binding‐protein‐1 (4EBP1) restored autophagy flux and reduced protein aggregates in myofiber‐specific knockout of TSC1 (TSC1mKO) mouse muscle. (A) Immunoblotting (left) and normalized quantification of p62 and ubiquitin immunoblotting (right) shown in graph as *n* = 4 mice per genotype from *Figures* *5A* and *S5B* in insoluble fraction from quadriceps muscle (QM) protein lysate of 12‐month‐old male mice. Ponceau S membrane staining was used as a loading control shown in *Figure* *S5A* and the accompanied blots with additional samples are shown in *Figure* *S5B*. The detailed quantification of *Figures* *5* *A* and *S5B* is presented in *Table* *S5*. *n* = 4 mice per genotype. Data are shown as mean ± SEM and individual points correspond to one mouse. Statistical significance was determined by one‐way ANOVA with Tukey's multiple comparison test: *P < 0.05 and ***P < 0.001. (B) Immunoblotting of muscle incubated in the presence or absence of NH4Cl and leupeptin (Leu) to inhibit lysosomal degradation from 12‐month‐old male mice following 48‐h fasting. Western blot shown is representative of three separate blots shown in *Figure* *S5E*. The detailed quantification is shown in *Table* *S7*. (C) The quantification of LC3‐II formation from *Figures* *5B* and *S5E*. Proteins of interest were quantified relative to total protein by Ponceau S staining. Data are shown as individual points that correspond to one mouse. ^$^ *P* < 0.05 indicates significant difference in LC3‐II expression between inhibitor treatment conditions by paired *t*‐test in control mice, whereas statistical analysis for 4EBP1mt‐TSC1mKO mice is 0.08 (colour indicates genotype). (D) Immunoblotting of autophagy markers from 12‐month‐old male mouse QM following 48‐h fasting; Ponceau S membrane staining was used as a loading control shown in *Figure* *S5D*. The quantification is shown in *Table* *S6*. (E) Immunoblotting of autophagy markers during prolonged fasting intervals in 12‐month‐old male mouse QM. Western blot shown is representative of four separate blots shown in *Figure* *S5F*. (F) Normalized quantification of LC3‐II/LC‐I ratio shown in graph as *n* = 4 mice per genotype per time points from *Figures* *5E* and *S5F*. Data are shown as mean ± SEM and individual points correspond to one mouse. Statistical significance was determined by two‐way ANOVA with Tukey's multiple comparison test: ^#^ *P* < 0.05, ^##^ *P* < 0.01 and ^###^ *P* < 0.001.

Up‐regulation of autophagy–lysosomal gene expression could be due to the up‐regulation of FoxO3 signalling as previously reported.^9^ Yet, impaired protein degradation from chronic mTORC1 activation was observed (*Figure* *5* *A*). mTORC1‐mediated suppression of autophagosome formation through direct phosphorylation of ULK has been shown to drive autophagy inhibition, leading to protein aggregate accumulation.^9^ Consistently, in the present study, mTORC1‐mediated phosphorylation of ULK at Ser757 and accumulation of the autophagosome cargo protein p62 were detected at high levels in TSC1mKO mouse muscles, which is correlated with increased protein ubiquitination, characteristic of protein aggregation. 4EBP1 activation significantly reduced levels of p62 and ubiquitinated protein in the TSC1mKO background, although increased mTORC1‐mediated ULK1 phosphorylation was still present (*Figures* *5* *A,D* and *S5C*).

To further validate the defective autophagosome formation, lysosome inhibitors were applied to block lysosomal degradation ex vivo, and the accumulation of lipidated LC3 (LC3‐II) was used to quantify autophagosome formation.^25^ An increase in LC3‐II with lysosome inhibition was undetectable in TSC1mKO mice, whereas 4EBP1mt‐TSC1mKO mice partially restored the treatment‐induced LC3‐II formation (*Figure* *5* *B,C*).

Upon autophagy stimulation (e.g., by starvation), a decrease in LC3‐I with a corresponding increase in LC3‐II protein levels indicates autophagosome formation. In 4EBP1mt‐TSC1mKO mouse muscle, the LC3‐II:LC3‐I ratio increased in response to starvation in a similar manner to control mice (*Figure* *5* *E,F*), reflecting autophagic flux changes in a nutrient‐sensitive manner. In comparison, only a small non‐significant change in the LC3‐II:LC3‐I ratio with prolong fasting was detected in TSC1mKO mice. Taken together, these data support the notion of impaired autophagy in TSC1mKO mice that is rescued by 4EBP1 activation.

### Myofiber activation of 4EBP1 rescues lysosomal dysfunction in TSC1mKO mouse muscle

**Figure 6:**
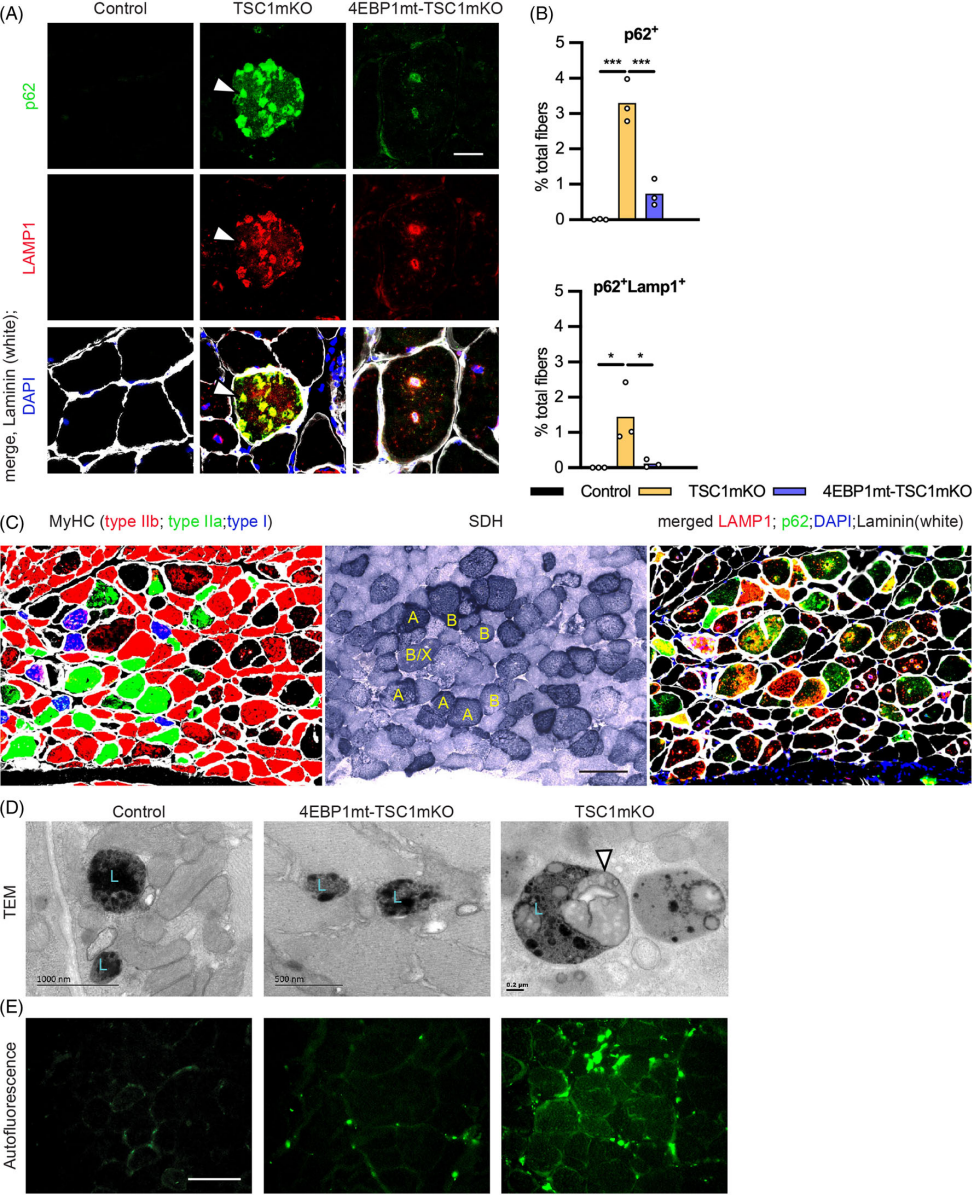
Myofiber activation of eIF4E‐binding‐protein‐1 (4EBP1) attenuated the accumulation of autophagy–lysosome aggregates in myofiber‐specific knockout of TSC1 (TSC1mKO) mouse muscle. (A) Confocal imaging of immunofluorescent staining of p62 and LAMP1 on cross‐sections of 12‐month‐old male gastrocnemius muscle (GM). DAPI staining was used to label nuclei and Laminin staining was used to outline the myofiber. Arrow heads indicate puncta with colocalized staining of p62 and LAMP1. Scale bar, 20 μm. (B) Quantification of immunofluorescence (IF) staining by positive staining in fibres from TissueFAXS images shown in *Figure* *S6B*. *n* = 3 mice per genotype. Data are shown as mean ± SEM and individual points correspond to one mouse in graphs. Statistical significance was determined by one‐way ANOVA with Tukey's multiple comparison test: *P < 0.05 and ***P < 0.001. (C) Colocalization analysis of myosin heavy chain (MyHC), succinate dehydrogenase (SDH) and autophagy markers staining in 12‐month‐old male TSC1mKO mouse GM. Indicated muscle type with LAMP1, p62 or cytoplasmic accumulation of both is labelled in the SDH image. Scale bar, 100 μm. (D) Transmission electron microscopy (TEM) imaging of soleus muscle of 12‐month‐old male mice fasted for 18 h; lysosomes (labelled ‘L’) and lipofuscin (white arrowheads) are indicated. (E) Autofluorescence is shown by excitation at 488 nm (green) in 12‐month‐old male quadriceps muscle (QM), representative for *n* = 3 mice per genotype. Scale bar, 20 μm.

In agreement with previous findings,^9^ we found that p62 accumulated as giant aggregates in the cytoplasm of TSC1mKO mouse myofibers. Moreover, the majority of myofibers with p62 aggregates also co‐stained with LAMP1 in our study (*Figures* *6* *A,B* and *S6A*). Analysis of serial‐stained sections revealed that LAMP1 staining was detected in all fibre types, whereas p62 was mainly detected in type II fibres (*Figures* *6* *C* and *S6B,C*). Myofibers that contained both LAMP1 and p62 aggregates were usually large and rounded or small and angular in shape and stained strongly with SDH (*Figure* *6* *C*), suggesting concurrent accumulation of mitochondria. Existence of LAMP1 aggregates in myofibers of TSC1mKO mice suggests impaired lysosomal degradation, supported by the presence of inclusions (*Figure* *S3E*) and glycogen accumulation (*Figure* *2* *E*) in muscle, characteristic of lysosome storage disease.^26^, ^27^ Ultrastructural analysis also revealed that lipofuscin accumulated inside lysosomes in TSC1mKO (*Figure* *6* *D*). Lipofuscin is a product of highly oxidized proteins, lipids, sugars and metals that cannot be degraded by proteasomes or eliminated by exocytosis and thus accumulates in post‐mitotic cells with age.^28^, ^29^ Lipofuscin is autofluorescent in nature as shown by increased autofluorescence at fibre boundaries and in the cytoplasm of select myofibers in TSC1mKO mice (*Figure* *6* *E*). These findings indicate that defective lysosomal degradation might further exacerbate protein aggregation from impaired autophagy, aggravating myopathy and leading to type II fibre degeneration in TSC1mKO mice.

Conversely, activation of 4EBP1 eliminated p62–LAMP1‐positive aggregates in the TSC1mKO mouse background, where the majority of the LAMP1 signal was detected around central nuclei or appeared as distinct puncta in 4EBP1mt‐TSC1mKO mice (*Figures* *6* *A,B* and *S6A*). Furthermore, lipofuscin was absent in lysosomes and autofluorescence was reduced in 4EBP1mt‐TSC1mKO mice (*Figure* *6* *D,E*).

**Figure 7:**
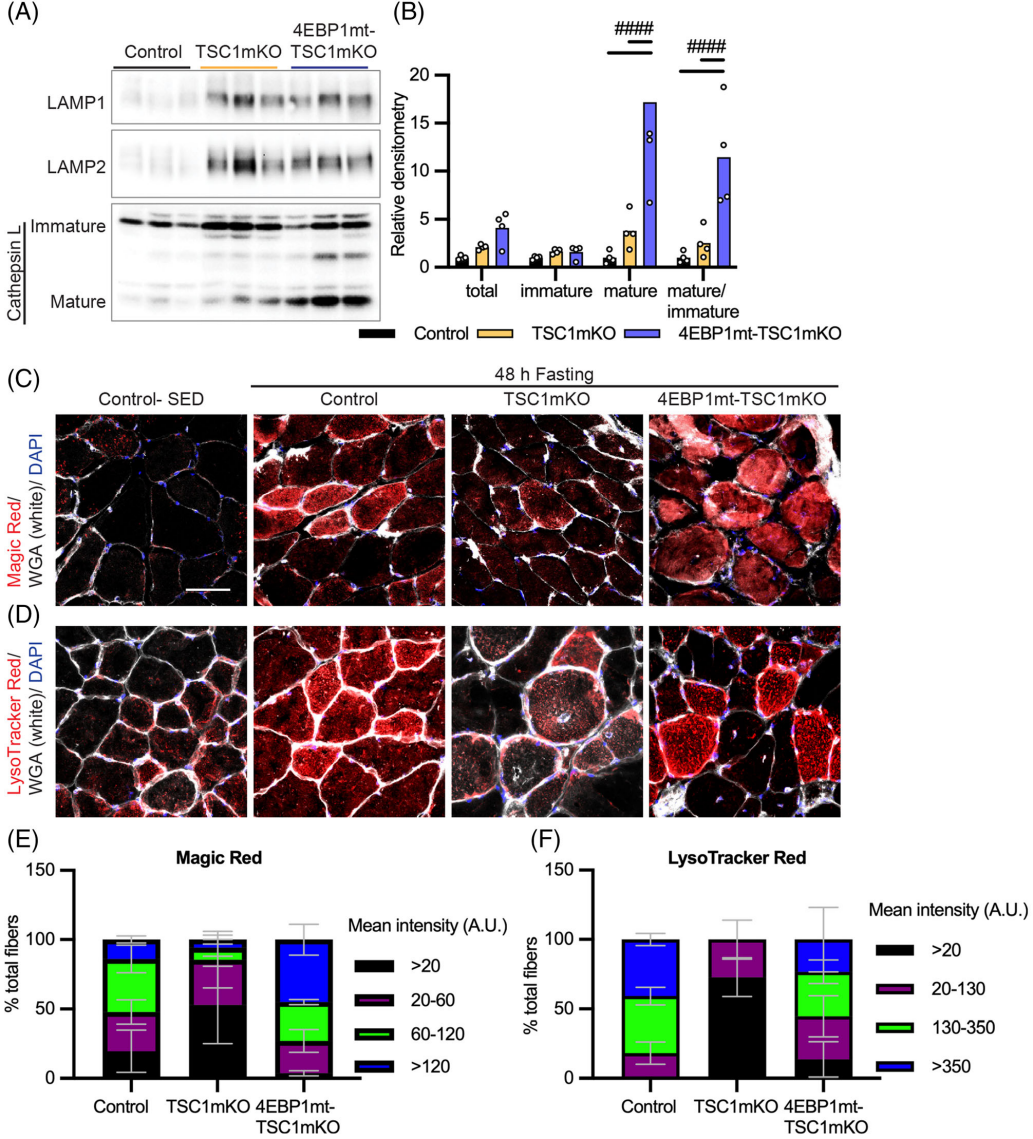
Myofiber activation of eIF4E‐binding‐protein‐1 (4EBP1) enhanced lysosomal protease activities in myofiber‐specific knockout of TSC1 (TSC1mKO) mouse muscle. (A) Immunoblotting of lysosomal markers in 12‐month‐old male mouse muscle following 48‐h fasting. Ponceau S was used as a loading control shown in *Figure* *S7A* and the accompanied blots with additional samples are shown in *Figure* *S7B*. The western blot quantification is shown in *Table* *S6*. (B) The quantification of Cathepsin L from *Figures* *7A* and *S7B*. Data are shown as mean ± SEM and individual points correspond to one mouse. Statistical significance was determined by two‐way ANOVA with Tukey's multiple comparison test: ^####^ *P* < 0.0001. (C) Magic Red and (D) LysoTracker Red staining of gastrocnemius muscle (GM) of 12‐month‐old male mice. Scale bar, 50 μm. Images for 48‐h fasting mice are representative for *n* = 3 mice per genotype. (E) Quantification of Magic Red staining and (F) LysoTracker Red staining by intensity class. The statistical analysis of quantification of *Figure* *7E* is present in *Table* *S8* and *Figure* *7F* is present in *Table* *S9*.

Next, we investigated the biochemical properties of these lysosomes. Accumulation of lysosomal membrane markers LAMP1 and LAMP2 as well as enzyme Cathepsin L were confirmed at the protein level by western blot analysis (*Figure* *7* *A*). Lysosomal hydrolases are predominantly composed of cathepsin enzymes, among which Cathepsin L was up‐regulated in all genetic groups compared with control mice (*Figure* *7* *A,B*). Although up‐regulated cathepsin expression was likely due to increased lysosomal content, mature Cathepsin L (active form) was not correspondingly increased in TSC1mKO muscle, whereas 4EBP1mt‐TSC1mKO mice showed up‐regulated mature Cathepsin L expression, indicating enhanced lysosomal hydrolase activity. To further validate cathepsin activity in vivo, Magic Red staining was performed in muscle. Magic Red is composed of Cathepsin B and L substrates that fluoresce red upon cleavage by active cathepsins. Magic Red staining was significantly decreased in TSC1mKO mouse muscle, which was restored by 4EBP1 activation, consistent with higher mature Cathepsin L expression (*Figure* *7* *A–C,E*).

The conversion of immature to mature Cathepsin L is processed at pH 3.0–6.5 in lysosomes, where it functions as a protease. Therefore, we hypothesized that intralysosomal pH changes in TSC1mKO mouse muscle may be the cause of the cathepsin maturation defect. To test this possibility, we examined lysosomal pH using LysoTracker Red, a lysosomal acidity indicator that displays higher fluorescence in more acidic environments.^30^, ^31^ LysoTracker Red staining was first validated in the muscle of control mice in fed versus 48‐h starvation conditions. In concordance with previous findings that nutrient starvation induces acidity in the lysosome,^32^ LysoTracker Red intensity was elevated in muscle of control mice and 4EBP1mt‐TSC1mKO mice following 48‐h fasting (*Figure* *7* *D,F*). In 48‐h‐starved TSC1mKO mice, LysoTracker Red fluorescence in muscle was significantly reduced. LysoSensor Green staining was performed to further confirm that lysosomal pH in TSC1mKO muscle was indeed elevated (*Figure* *S7D*). These findings supported our hypothesis that defective cathepsin enzyme activity was due to elevated lysosomal pH.

**Figure 8:**
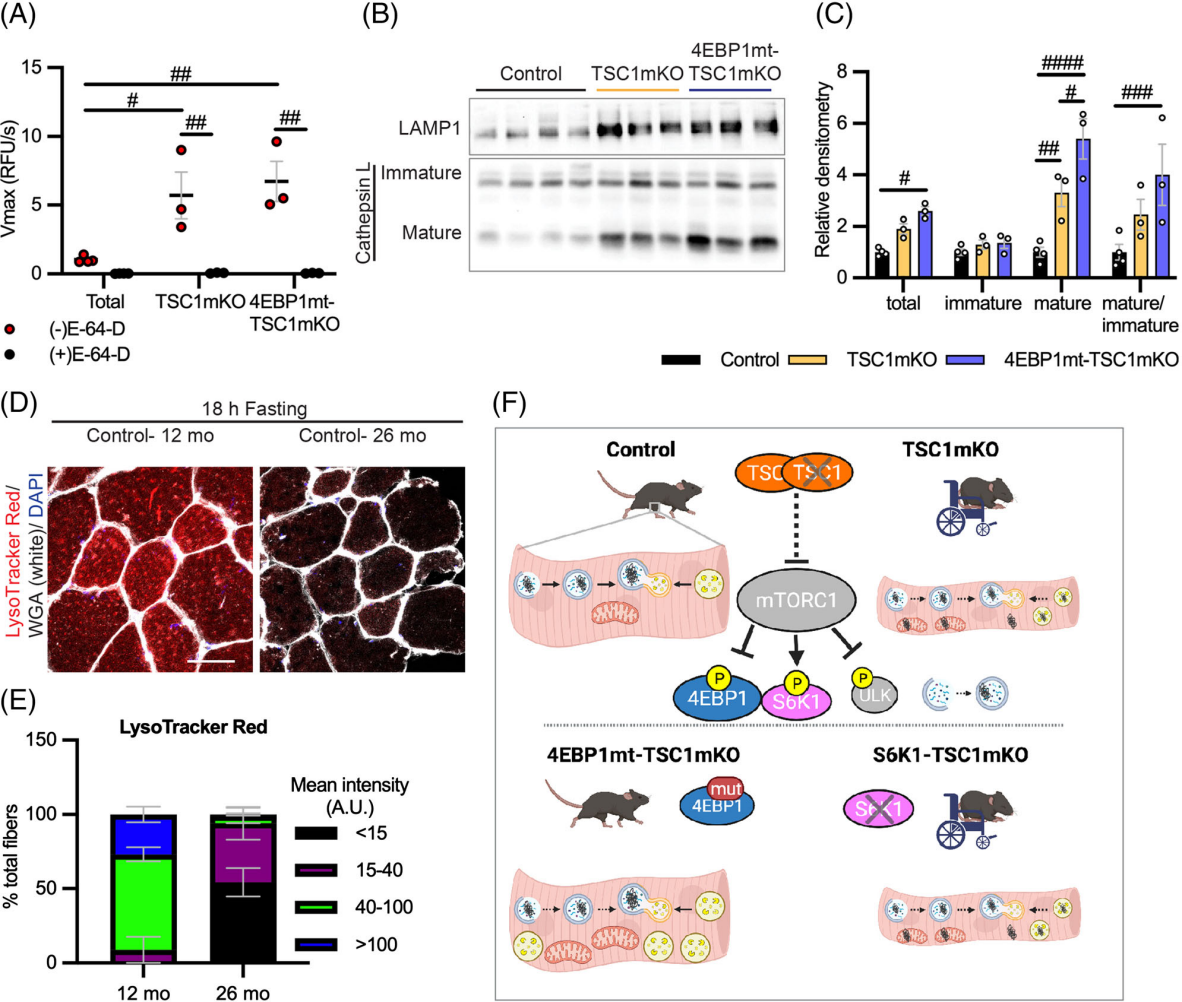
Lysosomal dysfunction was conserved in the natural aging mouse muscles. (A) Cathepsin activity assay performed in muscle acid lysate (MAL) from 12‐month‐old male mice following 18‐h fasting (control *n* = 4, TSC1mKO *n* = 3, 4EBP1mt‐TSC1mKO *n* = 3). Samples were treated with E‐64‐D as an inhibitor of cathepsin activity or with cathepsin assay buffer (untreated group). (B) Immunoblotting of MAL from 12‐month‐old male mice following 18‐h fasting. Coomassie blue staining was used to demonstrate equal loading and Ponceau S was used for protein quantification shown in *Figure* *S7C* and the quantification is shown in *Table* *S10*. (C) Quantification is shown for total Cathepsin L expression and ratio between mature and immature Cathepsin L from *Figure* *8B* (control *n* = 4, TSC1mKO *n* = 3, 4EBP1mt‐TSC1mKO *n* = 3). Data are shown as mean ± SEM and individual points correspond to one mouse. Statistical significance was determined by two‐way ANOVA with Tukey's multiple comparison test: ^#^ *P* < 0.05, ^##^ *P* < 0.01, ^###^ *P* < 0.001 and ^####^ *P* < 0.0001 indicate statistical significance. (D) Representative image of LysoTracker Red staining from control male mouse quadriceps muscle (QM) at the different age. Scale bar, 50 μm. Images are representative of *n* = 3 mice per genotype. (E) Quantification of LysoTracker Red staining by intensity class from *n* = 3 mice per genotype. The quantification of *Figure* *8E* is in *Table* *S11*. (F) Graphical summary of research. The blue and yellow vesicles indicate autophagosome and lysosome, respectively. Hyperactivation of mTORC1 suppresses the autophagosome formation, subsequently inducing protein aggregate formation in the cytosol and causing the dysfunction of mitochondria and lysosomes. As a consequence, myofiber‐specific knockout of TSC1 (TSC1mKO) accelerates sarcopenia development. Myofiber‐specific activation of eIF4E‐binding‐protein‐1 (4EBP1), but not inactivation of S6K1, restores muscle strength and function and reduces proteotoxic stress by lowering aberrant protein synthesis and enhancing lysosomal protease activities to compensate for impaired autophagy driven by mTORC1 hyperactivation.

To provide further evidence that defective lysosomal acidification was the major cause of reduced cathepsin activity and thus lysosome dysfunction in TSC1mKO muscle, we examined cathepsin activity in acidified muscle lysate in vitro. In this acidic condition, cathepsin activity was elevated comparably in TSC1mKO and 4EBP1mt‐TSC1mKO muscles (*Figure* *8* *A*), and levels of mature Cathepsin L isoform were similar (*Figure* *8* *B,C*). These data indicate that cathepsins can be processed correctly when the environment is sufficiently acidic.

Taken together, our data show that sustained mTORC1 activation impairs lysosomal acidification that in turn inhibits cathepsin maturation and lysosomal degradation in muscle. To determine whether lysosomal dysfunction also occurs in the naturally aged mouse muscle, in which mTORC1 activity is dysregulated, we compared LysoTracker Red staining in muscles of aged (26‐month‐old) and adult (12‐month‐old) wildtype mice following overnight fasting. Similar to TSC1mKO mice, the intensity of LysoTracker Red staining was significantly reduced in aged mice compared with their younger counterparts (*Figure* *8* *D,E*), indicating that impaired lysosomal acidification is a common phenotype in mTORC1‐driven sarcopenia.

## Discussion

mTORC1 is hyperactive in aging human and mouse skeletal muscle,^5^, ^10^ where it is insensitive to nutrient and mechanical signals, perturbing proteostasis. The present study demonstrates that 4EBP1 activation could rescue mTORC1‐induced sarcopenia, improving muscle size and strength in fast‐twitch muscle. We identify that activation of 4EBP1 resets the basal protein synthesis rate and expands lysosomal degradation capacity to compensate for the impaired autophagy caused by mTORC1 hyperactivation. Activation of 4EBP1 preserved lysosomal acidity and increased the number of functional lysosomes with elevated cathepsin activity, relieving proteotoxic stress resulting from the accumulation of damaged proteins and mitochondria to prevent muscle functional decline in TSC1mKO mouse background (*Figure* *8* *F*).

In line with our previous findings, an up‐regulation of autophagy activities was observed in 4EBP1‐activated myofibers and counteracted aging‐associated muscle atrophy.^14^ It is apparent that maintained or slightly excessive proteolysis could preserve muscle mass with age, rather than increasing protein synthesis, as observed in TSC1mKO mice. Currently, the target of 4EBP1 in regulating autophagy–lysosome function is unclear. In yeast, 4EBP Eap1 regulates translation of autophagy–lysosome‐related genes, inhibiting cap‐dependent translation in a similar manner to 4EBP1.^33^ Furthermore, Eap1 was recently shown to interact with Dhh1, a DExD/H‐box RNA helicase, to facilitate the translation of *Atg1* and *Atg13* under nitrogen‐starvation conditions.^34^ Similarly, 4EBP1 has been proposed to be responsible for dietary restriction‐induced mRNA translation of mitochondrial genes in *Drosophila*, which mediates lifespan extension.^35^ These studies suggest that 4EBP1 could differentially enhance the translation of certain genes during nutrient limitation and could potentially override inhibition of the autophagy–lysosome system in conditions of mTORC1 hyperactivation. Future investigations using techniques such as ribosomal profiling (Ribo‐seq) or ribosomal tagging (Ribo‐Tag mice) are warranted for translatome analysis in skeletal muscle to validate the translational targets of 4EBP1.

Repression of the PI3K/AKT/mTORC1/S6K signalling axis such as by rapamycin treatment or calorie restriction is known to extend lifespan and promote healthspan across model organisms.^36^ Furthermore, inactivation of S6K1 in muscle has been shown to extend lifespan and improve endurance running performance in the *Lmna* knockout background (S6K1^f/f^;Ckmm‐Cre;Lmna^−/−^), similar to rapamycin treatment.^16^ Even though there were no direct measurements of muscle mass and strength reported in aging *S6k1* whole‐body knockout mice or muscle‐specific S6K1 knockout in the *Lmna* whole‐body knockout background compared with their aged match control (*wildtype*) or *Lmna* whole‐body knockout mice, respectively, improved endurance running performance with enhanced mitochondrial biogenesis was shown in both studies.^20^, ^37^ In TSC1mKO mice, even short‐term rapamycin treatment could restore myofiber size and force generation to control levels.^9^, ^38^ Thus, it was surprising that the contribution of S6K1 to myopathic effects was negligible in the TSC1mKO background. Despite reduced global translation, high proteotoxic stress from impaired autophagy and elevated cap‐dependent translation resulted in myopathy, observed in S6K1‐TSC1mKO mouse muscle. We could not exclude the compensatory effect from S6K2 contributing to the myopathy in S6K1‐TSC1mKO mice, yet a similar phenomenon has been observed in AKT‐stimulated muscle hypertrophy, where S6K1 inactivation did not limit cellular growth but exacerbated the accumulation of p62 aggregates and decreased force production in muscle with AKT hyperactivation (TgAkt‐S6K1KO mice).^39^ Notably, compensatory up‐regulation of 4EBP1 phosphorylation in TgAkt‐S6K1KO mice was reported,^39^ which is likely to impede autophagic activity in a similar manner to what we observed in S6K1‐TSC1mKO mouse muscle. This functional feedback loop links mTORC1 downstream targets in the regulation of protein synthesis. Thus, caution is warranted with treatments inhibiting S6K1 in conditions of mTORC1 hyperactivation, which may block cellular activities dependent on 4EBP1 inhibition.

Overall, we reveal a previously unidentified phenotype of lysosomal dysfunction caused by mTORC1 hyperactivation through 4EBP1 in muscle. Our study provides new evidence that activation of the autophagy–lysosome system by specifically targeting 4EBP1 could restore myofiber proteostasis and function. These findings suggest that blockade of autophagic flux by mTORC1 can be bypassed by 4EBP1 activation, restoring nutrient sensitivity, as a therapeutic avenue for sarcopenia treatment.

## Conflict of interest

No potential competing interest was reported by the authors.

## Supporting information


**Table S1.** Transgenic mouse lines used in this study.
**Table S2.** Reagents and antibodies used in this study.
**Table S3.** qPCR primers used in this study.
**Table S4.** Quantification of western blot analysis in QM protein lysate from 12‐mo‐old male mice, related to Figure 3A, S2B, S3I, and S5C.
**Table S5.** Quantification of western blot analysis in QM insoluble protein fraction from 12‐mo‐old male mice, related to Figure 5A and S5B.
**Table S6.** Quantification of western blot analysis in QM protein lysate from 12‐mo‐old male mice following 48 h fasting, related to Figure 5D, 7A and S7B.
**Table S7.** Quantification of western blot analysis of QM with/without lysosome inhibitors treatment ex vivo from 12‐mo‐old male mice following 48 h fasting, related to Figure 5B and S5E.
**Table S8.** Quantification of Magic Red staining in GM from 12‐mo‐old male mice following 48 h fasting, related to Figure 7E.
**Table S9.** Quantification of Lysotracker Red staining in GM from 12‐mo‐old male mice following 48 h fasting, related to Figure 7F.
**Table S10.** Quantification of western blot analysis in QM muscle acid lysate from 12‐mo‐old male mice following 18 h fasting, related to Figure 8B.
**Table S11.** Quantification of Lysotracker Red staining in QM from control male mice following 18 h fasting, related to Figure 8E.
**Figure S1.** Representative confocal images of immunofluorescence staining on negative control were taken from TSC1mKO mouse quadriceps muscle.
**Figure S2.** Molecular and physiological characterization of TSC1mKO, S6K1‐TSC1mKO and 4EBP1mt‐TSC1mKO mice.
**Figure S3.** 4EBP1 activation ameliorated sarcopenic pathology in TSC1mKO mouse muscle.
**Figure S4.** RNA‐seq analysis of transcriptome and translatome from 2‐mo‐old TSC1mKO male mouse skeletal muscle.
**Figure S5.** The loading control and accompany immunoblotting images for Figure 5.
**Figure S6.** Activation of 4EBP1 lowered proteostatic stress in TSC1mKO mouse muscle.
**Figure S7.** Lysosomal pH is elevated in TSC1mKO mouse muscle.
