# Supplementary material for: Activation of eIF4E‐binding‐protein‐1 rescues mTORC1‐induced sarcopenia by expanding lysosomal degradation capacity
Source: J Cachexia Sarcopenia Muscle. 2022 Nov 17;14(1):198–213. doi: 10.1002/jcsm.13121 (PMC9891956; doi:10.1002/jcsm.13121)
Supplement: Supplementary file 1 — Table S1. Transgenic mouse lines used in this study. Table S2. Reagents and antibodies used in this study. Table S3. qPCR primers used in this study. Table S4. Quantification of western blot analysis in QM protein lysate from 12‐mo‐old male mice, related to Figure 3A, S2B, S3I, and S5C. Table S5. Quantification of western blot analysis in QM insoluble protein fraction from 12‐mo‐old male mice, related to Figure 5A and S5B. Table S6. Quantification of western blot analysis in QM protein lysate from 12‐mo‐old male mice following 48 h fasting, related to Figure 5D, 7A and S7B. Table S7. Quantification of western blot analysis of QM with/without lysosome inhibitors treatment ex vivo from 12‐mo‐old male mice following 48 h fasting, related to Figure 5B and S5E. Table S8. Quantification of Magic Red staining in GM from 12‐mo‐old male mice following 48 h fasting, related to Figure 7E. Table S9. Quantification of Lysotracker Red staining in GM from 12‐mo‐old male mice following 48 h fasting, related to Figure 7F. Table S10. Quantification of western blot analysis in QM muscle acid lysate from 12‐mo‐old male mice following 18 h fasting, related to Figure 8B. Table S11. Quantification of Lysotracker Red staining in QM from control male mice following 18 h fasting, related to Figure 8E. Figure S1. Representative confocal images of immunofluorescence staining on negative control were taken from TSC1mKO mouse quadriceps muscle. Figure S2. Molecular and physiological characterization of TSC1mKO, S6K1‐TSC1mKO and 4EBP1mt‐TSC1mKO mice. Figure S3. 4EBP1 activation ameliorated sarcopenic pathology in TSC1mKO mouse muscle. Figure S4. RNA‐seq analysis of transcriptome and translatome from 2‐mo‐old TSC1mKO male mouse skeletal muscle. Figure S5. The loading control and accompany immunoblotting images for Figure 5. Figure S6. Activation of 4EBP1 lowered proteostatic stress in TSC1mKO mouse muscle. Figure S7. Lysosomal pH is elevated in TSC1mKO mouse muscle. [file JCSM-14-198-s001.pdf]

Supplemental Information

Title: Activation of eIF4E-binding-protein-1 rescues mTORC1-induced sarcopenia by expanding lysosomal degradation capacity.

Authors and affiliations:

Elisa M. Crombie[1], Seonyoung Kim[1], Stuart Adamson[3], Han Dong[1], Tzu-Chiao Lu[9], Yiju Wu[1], Yajun Wu[4], Yotam Levy [5], Nolan Stimple[3], Wing Moon R. Lam[6], Hwee Weng D. Hey[6], Dominic J. Withers[7], Ao-Lin Hsu[9, 10], Boon Huat Bay[4], Julien Ochala[5,8], Shih-Yin Tsai[1,2]

Supplemental Information titles and legends

Supplemental Materials and Methods

Supplemental Table S1-S11

Supplemental Reference

Supplemental Figure S1-S7

Supplemental Materials and Methods

Single myofiber force production

Cross-sectional area (CSA) and force of single membrane-permeabilized myofibers were analyzed as described previously [1].

Micro-computed tomography (micro-CT)

Following mouse sacrifice, scans of the thoracic and lumbar spine were performed at 18 µm pixel size cuts by SkyScan 1176 micro-CT (Bruker, Kontich, Belgium), equipped with a 0.5 mm aluminium filter at 50 kV and current of 500 uA. Sagittal spinal alignment was measured using the Cobb method on ImageJ software.

Metabolic cage

Oxygen consumption was measured by open-flow respirometry (Sable Systems) as previously described [2].

Ex vivo autophagic flux assay

The lysosome inhibitor assay was performed with a protocol kindly provided by Ana Maria Cuervo [3] with some modifications. Muscle from 48-h-fasted mice were minced and incubated with or without lysosome inhibitors NH_4Cl and Leupeptin in 2 ml DMEM at 37°C in a 5% CO_2 incubator for 2 h with occasional swirling. After the incubation, the tissue/medium suspension was collected and centrifuged at 1000 ×g for 5 min at 4°C. The tissue was washed twice with ice-cold PBS. After washing, tissue was homogenized in 500 µl of 0.25 M sucrose supplemented with protease inhibitors and sonicated for 5 s on the high setting. Homogenates were then analyzed as described above for western blot analysis.

Mitochondrial isolation and seahorse assay

Mitochondria were isolated from 12-mo-old female mice and analyzed in XF24 Extracellular Flux Analyzer (Seahorse Bioscience) as previously described [2] with following modifications. Extracellular flux was analyzed in the presence of succinate/rotenone (1.5 μg mitos), glutamate/malate (3 μg mitos), pyruvate/malate (3 μg mitos), or palmitoyl carnitine/malate (5 μg mitos). Each analysis was performed with 5 replicates per substrate. OCR was normalized to TSC1mKO as the plate control group to account for day-to-day variation in mitochondrial isolation and Seahorse conditions (n=4 mice/genotype).

Polysome profiling

Polysome profiling was modified from previous published work [4-6] . Briefly, limb muscles from 2-mo-old male mice were homogenized with a Dounce homogenizer in ice-cold lysis buffer [5] and the homogenate was then centrifuged at 5,000 ×g for 15 min at 4°C. The supernatant from centrifugation was measured at OD260 and equal volumes of samples were layered on the top of a pre-cooled 15-60% sucrose gradient [6]. Samples were then centrifuged in a SW41Ti rotor at 39,000 rpm for 4 h at 4°C. Gradients were collected from the top and profiles were monitored at 254 nm.

RNA-seq data processing

Fastq files of transcriptome and translatome data were trimmed for removing adapters using Trim Galore version 0.6.7 (https://doi.org/10.5281/zenodo.5127899). Trimmed reads were mapped to the mouse genome (mm10) using STAR version 2.7.1a [7]. Expression of each gene was measured using featureCount function from Subread version 1.6.4 [8].

Differential expression analysis

Genes differentially expressed in each genotype were identified using DESeq2 version 1.26.0 [9]. Genes with reads less than 10 were removed for the DESeq2 analysis. Genes with false

discovery rate (FDR)-adjusted P value < 0.05 and fold change ≧ 1.5 were considered to be differentially expressed. Variance stabilizing transformation (VST) was applied to the gene counts before the principal component analysis (PCA).

Venn diagram and gene ontology analysis

Venn diagrams were generated using matplotlib-venn version 0.11.6 (https://github.com/konstantint/matplotlib-venn). For each genotype, genes differentially expressed relative to the wild-type samples were examined for the enriched Gene Ontology (GO) terms using DAVID [10] or Metascape [11].

Immunohistochemistry and Immunofluorescence staining (IF)

Hematoxylin and eosin (H&E), Oil-Red-O, and muscle fiber type staining were performed as previously described [2]. For Periodic acid–Schiff (PAS) staining, a PAS staining kit was used for detection of aldehydes and mucosubstances, in concordance with the manufacturer's instructions. For immunohistochemistry of succinate dehydrogenase (SDH) activities, following defrosting, slides were then rehydrated in PBS for 5-min, then incubated in SDH solution for 2-3 min at room temperature (RT). Subsequently, slides were washed with PBS and mounted with aqueous mounting medium. For p62, LAMP1, and laminin IF staining, following defrosting, tissues were fixed in 4% PFA (10 min). Following with PBS washing, slides were boiled in Target Retrieval Solution for antigen retrieval (10 min), then left to cool at RT (10 min). After a washing step, slides were blocked at RT (1 hr) and then incubated with primary antibody at 4°C overnight. After washing with PBS, the secondary antibody solution was added and incubated (1 hr) at RT. Samples were subsequently washed with PBS and then mounted with Prolong Gold antifade reagent. In a separate experiment, muscle was stained with Wheat Germ Agglutinin (WGA) instead of laminin to outline the muscle fiber. For Dihydroethidium (DHE), LysoTracker

Red, LysoSensor Green and Magic Red staining, following defrosting, slides were washed with PBS. The fluorescence probes were then added on the slides (10 min) in a dark chamber. All the staining solution contained DAPI and WGA. The reaction was stopped by three washes in PBS. Slides were mounted in Prolong Gold antifade reagent.

Slides were imaged at 20× magnification with stitching into whole sections using a TissueFAXS Slide Scanner (TissueGnostics), with additional viewing at high magnification (60x) using an Olympus FV3000 Confocal Microscope. Serial unstained sections for IF or fluorescence probe with only DAPI and WGA was imaged as a negative control (**Figure S1**). For fiber type staining, whole tissue sections were analyzed to determine myofiber size of MyHC isoform populations using the Myosoft plugin of Fiji according to published methods [12]. For other IF staining, staining intensity per myofiber was determined by thresholding in ImageJ and fibers were also counted manually to determine number of positively stained fibers.

To analyse autofluorescence, following defrosting, tissues were then fixed in 2% PFA for 10 min. After a washing step, tissues were mounted with Antifade Mounting Medium. Slides were imaged using an Olympus DP72 upright microscope. Autofluorescence intensity was determined by mean fluorescence of five 20X images using ImageJ.

Table S1. Transgenic mouse lines used in this study.

Mouse line	Source	Reference
Ckmm-Cre	Jackson Lab. 006475	[13, 14]
TSC1flox	Jackson Lab. 005680	[15]
4EBP1mt	Our lab	[2]
S6K1flox	Dominic J Wither’s lab	[16]

Table S2. Reagents and antibodies used in this study.

Reagents	Source	Catalogue no	Working con.
Puromycin	Invitrogen	53-79-2	0.04 µM/g bodyweight
Bicinchoninic Acid (BCA) Protein Assay	Pierce	23225	
Ponceau S	Sigma	P3504	0.1%
Protease Inhibitor Cocktail	Roche	04693124001	1:1000
Phosphatase Inhibitor Cocktail II	Sigma-Aldrich	P5726	1:1000
Phosphatase Inhibitor Cocktail III	Sigma-Aldrich	P0044	1:1000
Pierce™ ECL Western Blotting Substrate	Pierce	32106	1:1000
Amersham ECL Prime Western Blotting Detection Reagent	Amersham	RPN2232	
m7GTP-agarose	Jena Bioscience	AC-155S	
lysosome inhibitor- NH4Cl	MP Biomedicals	#12125-02-9	20 mM
lysosome inhibitor- Leupeptin	Sigma-Aldrich	L2884	200 µM
DMEM	Sigma-Aldrich	D5030	
succinate	Sigma-Aldrich	Sigma-Aldrich	5 mM
rotenone	Sigma-Aldrich	83-79-4	20 nM
Malic acid	Sigma-Aldrich	M6413-25G	0.5 mM
Glutamic Acid	Sigma-Aldrich	G-1626-100G	5 mM
Sodium pyruvate	Sigma-Aldrich	113-24-6	10 mM
Palmitoyl-L-carnitine chloride	Sigma-Aldrich	P1645-5MG	60 µM
Cathepsin inhibitor E-64-D	Enzo Life Sciences	BML-PI107	50 µM
Z-FR-AMC fluorogenic substrate	Enzo Life Sciences	BML-P139-0010	40 µM
PFA	MP BIOMEDICALS	30525-89-4	4%

Hematoxylin Solution, Gill No. 1	Sigma-Aldrich	GHS132	
Eosin	Sigma-Aldrich	HT110232	
Oil Red O	Supelco	O9755	300 mg/dl
PAS staining kit	Merck	#1.01646.001	
VectaMount AQ Aqueous Mounting Medium	Vector Laboratories	H-5501	
Prolong Gold antifade reagent	Invitrogen	P36930	
VECTASHIELD® Antifade Mounting Medium	Vector Laboratories	H-1000	
Target Retrieval Solution	Dako	S1699	
Dihydroethidium staining solution	Invitrogen	D23107	10 μM
LysoTracker Red DND-99	Invitrogen	L7528	1:1000
LysoSensor Green DND-189	Invitrogen	L7535	1:1000
Magic Red staining solution of Cathepsin B and L substrates	Sigma	CS0370	1:100
WGA Alexa Fluor™ 647 Conjugate	Invitrogen	W32466	1 μg/ml
DAPI (4',6-Diamidine-2'-phenylindole dihydrochloride)	ROCHE	10236276001	1 mg/ml

**Buffers**	**Composition**
0.1% SDS RIPA lysis buffer	50 mM Tris pH 8, 150 mM NaCl, 0.5% sodium deoxycholate, 1% Triton X-100, 0.1% SDS
2% SDS RIPA lysis buffer	50 mM Tris pH 8, 150 mM NaCl, 0.5% sodium deoxycholate, 1% Triton X-100, 2% SDS
Buffer A for Cap (m^7GTP) Pull-Down Assay	10 mM Tris, pH 7.5, 150 mM KCl, 4 mM MgCl2, and 1 mM EDTA and 1% NP-40
Muscle acid lysis buffer	200 mM Na-acetate pH 5, 50 mM NaCl, 0.1% Triton X-100
Dehydrogenase (SDH) solution	1.5 mM Nitroblue tetrazolium, 130 mM Sodium succinate, 0.2 mM Phenazine methosulphate, and 0.1mM Sodium azide
IF blocking solution	3% BSA; 0.1% Tween-20 in PBS
TEM fixed solution	2% paraformaldehyde containing 3% glutaraldehyde in 0.1 M phosphate-buffer

Polysome lysis buffer [5]	1 g muscle per 1 mL; 0.25 M Sucrose, 50 mM HEPES, pH=7.4, 250 mM KCl, 5 mM $MgCl_2$, 0.1 mg/mL Cycloheximide, 1% Triton X-100, 0.4 µg/µl RNase inhibitor Ribo-Lock with a protease inhibitor cocktail tablet [Roche]
sucrose gradient buffer [6]	50 mM Tris-HCl, pH=7.4, 100 mM KCl, 15 mM $MgCl_2$, 0.1 mg/mL cycloheximide, 1 mg/mL Heparin

**Primary Antibody**	**Source**	**Catalogue no**	**Experiment**
LAMININ	Abcam	ab11575	IHC
MYH1	DSHB	6H1	IHC
MYH2	DSHB	SC-71	IHC
MYH4	DSHB	BF-F3	IHC
MYH7	DSHB	BA-F8	IHC
p62 / SQSTM1	Progen	GP62-C	IHC
LAMP1	DSHB	1D4B	IHC, WB
4E-BP1	Cell Signaling	9452L	WB
alpha-Actinin	Cell Signaling	3134S	WB
Cathepsin L	R&D	AF1515	WB
eIF4E	Cell Signaling	2067	WB
eIF4G	Cell Signaling	2469S	WB
GAPDH (14C10)	Cell Signaling	2118S	WB
HSP90 (C45G5)	Cell Signaling	4877S	WB
LAMP2	DSHB	ABL-93	WB
LC3B	Sigma	L7543	WB
OXPHOS Cocktail	Abcam	ab110411	WB
P-4E-BP1 (S65)	Cell Signaling	9451S	WB
P-4E-BP1 (Thr37/46)	Cell Signaling	2855S	WB
P-p70 S6 Kinase (T389) (108D2)	Cell Signaling	9234S	WB
P-S6 (Ser235/236)	Cell Signaling	2211S	WB
p-S6 (Ser240/244)	Cell Signaling	2215S	WB
p-S6K2 (pSer423)	Sigma	SAB4301562	WB
P-ULK1 (Ser757)	Cell Signaling	6888S	WB
p62 / SQSTM1	Abnova	H00008878-M01	WB
Puromycin	Sigma	MABE343	WB
S6 Ribosomal Protein (5G10)	Cell Signaling	2217S	WB
S6K1	Cell Signaling	2708L	WB
S6K2	Cell Signaling	14130	WB
TSC1	Axil Scientific	A300-316A	WB
UBIQUITIN	Cell Signaling	3933S	WB
ULK1	Sigma	A7481-200ul	WB

**Secondary Antibody**	**Source**	**Catalogue no**	**Experiment**

Goat anti-Mouse IgG2b Cross-Adsorbed Secondary Antibody, Alexa Fluor 350	Invitrogen	A21140	IHC
Goat anti-Mouse IgG1 Cross-Adsorbed Secondary Antibody, Alexa Fluor 488	Invitrogen	A21121	IHC
Goat anti-Mouse IgM (Heavy chain) Cross-Adsorbed Secondary Antibody, Alexa Fluor 594	Invitrogen	A21044	IHC
Goat anti-Rabbit IgG (H+L), Superclonal™ Recombinant Secondary Antibody, Alexa Fluor 647	Invitrogen	A27040	IHC
Goat anti-Guinea Pig IgG (H+L) Highly Cross-Adsorbed Secondary Antibody, Alexa Fluor 488	Invitrogen	A11073	IHC
Goat anti-Rat IgG (H+L) Cross-Adsorbed Secondary Antibody, Alexa Fluor 594	Invitrogen	A11007	IHC
Amersham ECL Mouse IgG, HRP-linked whole Ab (from sheep)	Cytiva	NA-931	WB
Amersham ECL Rabbit IgG, HRP-linked whole Ab (from donkey)	Cytiva	NA934	WB
Amersham ECL Rat IgG, HRP-linked whole antibody (from goat)	Cytiva	NA935	WB
Rabbit anti-Goat IgG (H+L) Secondary Antibody, HRP	Invitrogen	31402	WB

Table S3. qPCR primers used in this study.

Gene	Forward Primer	Reverse Primer
Sdhb	AATTTGCCATTTACCGATGGGA	AGCATCCAACACCATAGGTCC
Ndufa1	ATGTGGTTCGAGATTCTCCCT	TGGTACTGAACACGAGCAACT
Uqcrc2	AAAGTTGCCCCGAAGGTTAAA	GAGCATAGTTTTCCAGAGAAGCA
Cox2	ATAATCCCAACAAACGACCT	CTCGGTTATCAACTTCTAGCA
Ppia	GACCAAACACAAACGGTTCC	GGAAGGTGAAAGAAGGCATG

Table S4. Quantification of western blot analysis in QM protein lysate from 12-mo-old male mice, related to **Figure 3A, S2B, S3I,** and **S5C**.

	Control	TSC1mKO	4EBP1mt-TSC1mKO	S6K1-TSC1mKO
p-S6K1^{T389}	1 ± 0.48	21.6 ± 3.45**	22.9 ± 7.59***	1.02 ± 0.56$^{\#}$
S6K1	1 ± 0.15	0.58 ± 0.08**	0.51 ± 0.01***	0.08 ± 0.01****$^{\#\#\#}$
p-S6K2^{S423}	1 ± 0.18	1.72 ± 0.45	1.77 ± 0.12*	2.11 ± 0.68
S6K2	1 ± 0.10	0.60 ± 0.17	1.04 ± 0.19$^{\#}$	0.52 ± 0.16*
p-S6$^{S235/S236}$	1 ± 0.69	8.59 ± 2.26***	7.82 ± 0.77**	8.97 ± 1.23***
p-S6$^{S240/S244}$	1 ± 0.49	10.5 ± 5.69*	11.4 ± 2.45*	18.2 ± 3.03**
S6	1 ± 0.28	1.07 ± 0.21	1.48 ± 0.21	0.88 ± 0.08
p-4EBP1$^{T36/T47}$	1 ± 0.12	2.06 ± 0.11*	1.24 ± 0.27	1.52 ± 0.66
p-4EBP1^{S65}	1 ± 0.46	16.2 ± 1.27**	10.4 ± 5.13*	19.0 ± 2.87***
4EBP1	1 ± 0.46	2.36 ± 0.35	5.59 ± 0.72****$^{\#\#}$	4.01 ± 0.80**
eIF4E	1 ± 0.24	1.36 ± 0.10	2.55 ± 0.20****$^{\#\#\#\#}$	1.30 ± 0.02
p-ULK1^{S757}	1 ± 0.54	16.9 ± 8.31	40.3 ± 17.6*	25.7 ± 13.9
ULK1	1 ± 0.34	3.67 ± 2.10	3.24 ± 1.47	2.78 ± 1.21
p62	1 ± 0.48	6.56 ± 0.44****	3.79 ± 0.99**$^{\#\#}$	7.65 ± 0.32****
ATP5A	1 ± 0.15	2.50 ± 0.24***	2.34 ± 0.40**	2.52 ± 0.54***
UQCRC2	1 ± 0.29	3.84 ± 0.69	3.66 ± 1.47	3.97 ± 2.54
SDHB	1 ± 0.87	74.4 ± 85.2	22.9 ± 31.1	23.6 ± 18.4
COXII	1 ± 0.16	1.59 ± 0.58	1.63 ± 0.77	1.68 ± 0.56
NDUFB8	1 ± 0.35	1.52 ± 0.39	2.35 ± 0.57**	2.21 ± 0.53*

Relative protein changes are shown as mean ± SD, vs control mice. N=3-4 mice/genotype; each protein was analysed by one-way ANOVA with Tukey's multiple comparison test.

*P<0.05, **P<0.01, ***P<0.001, and ****P<0.0001 indicate significance vs control mice.
#P<0.05, ##P<0.01, ###P<0.001, and ####P<0.0001 indicate significance vs TSC1mKO mice.

Table S5. Quantification of western blot analysis in QM insoluble protein fraction from 12-mo-old male mice, related to **Figure 5A** and **S5B**.

	Control	TSC1mKO	4EBP1mt-TSC1mKO	
Ubiquitin	1 ± 0.14	2.42 ± 0.46***	1.58 ± 0.28[#]	
p62	1 ± 0.39	14.6 ± 4.14***	3.94 ± 1.68[###]	

Relative protein changes are shown as mean ± SD, vs control mice. N=4/genotype; each protein was analysed by one-way ANOVA with Tukey's multiple comparison test. ***P<0.001 indicates significance vs control mice. #P<0.05 and ###P<0.001 indicate significance vs TSC1mKO mice.

Table S6. Quantification of western blot analysis in QM protein lysate from 12-mo-old male mice following 48 h fasting, related to **Figure 5D, 7A** and **S7B**.

	Control	TSC1mKO	4EBP1mt-TSC1mKO
p-ULK1^{S757}	1 ± 1.16	39.8 ± 8.85**	44.0 ± 21.2**
ULK1	1 ± 0.85	12.2 ± 3.65**	6.91 ± 4.64
p62	1 ± 0.46	3.71 ± 0.63****	2.05 ± 0.68$^{\#\#}$
LAMP1	1 ± 0.34	3.77 ± 1.63*	4.11 ± 1.54*
LAMP2	1 ± 0.30	6.19 ± 1.30	10.2 ± 7.23*
Cathepsin L^{total}	1 ± 0.21	2.11 ± 0.27	4.11 ± 1.73**
Cathepsin L^{immature}	1 ± 0.17	1.65 ± 0.25	1.59 ± 0.69
Cathepsin L^{mature}	1 ± 0.63	3.80 ± 1.87	17.2 ± 12.2*

Relative protein changes are shown as mean ± SD, vs control mice. N=4-5/genotype; each protein was analysed by one-way ANOVA with Tukey's multiple comparison test. *P<0.05, **P<0.01, and ****P<0.0001 indicates significance vs control mice. ##P<0.01 indicates significance vs TSC1mKO mice.

Table S7. Quantification of western blot analysis of QM with/without lysosome inhibitors treatment ex vivo from 12-mo-old male mice following 48 h fasting, related to **Figure 5B** and **S5E**.

		**Control**	**TSC1mKO**	**4EBP1mt-TSC1mKO**
p62	Medium only	1	220.9 ± 384.8	184.2 ± 218.3
	NH_4Cl/Leu	132.7 ± 174.8	1165.2 ± 1331.5	469.3 ± 495.0
LC3-I	Medium only	1	23.3 ± 27.0	0.98 ± 0.58
	NH_4Cl/Leu	1.81 ± 1.45	17.4 ± 19.0	2.48 ± 2.80
LC3-II	Medium only	1	13.7 ± 10.3	3.17 ± 2.98
	NH_4Cl/Leu	5.97 ± 2.46*	19.9 ± 12.8	8.50 ± 4.20

Relative protein changes are shown as mean ± SD, relative to untreated (medium only) control mice. Control, N=3; TSC1mKO, N=5; 4EBP1mt-TSC1mKO, N=3 across three different blots. *P<0.05, analysed by paired t-test per genotype.

Table S8. Quantification of Magic Red staining in GM from 12-mo-old male mice following 48 h fasting, related to **Figure 7E**.

**Mean intensity (A.U.)**	**Control**	**TSC1mKO**	**4EBP1mt-TSC1mKO**
<20	19.4 ± 26.5	53.0 ± 48.4	3.54 ± 3.15[#]
20-60	28.4 ± 15.2	32.6 ± 35.3	23.4 ± 14.3
60-120	38.3 ± 17.0	8.52 ± 10.3	28.1 ± 3.16
>120	13.9 ± 4.39	28.1 ± 3.16	45.0 ± 19.1

Percentage of total fibers in each intensity class is shown as mean ± SD. N=3/genotype; data was analysed by two-way ANOVA with Tukey's multiple comparison test (across rows). #P<0.05 indicates significance vs TSC1mKO mice.

Table S9. Quantification of Lysotracker Red staining in GM from 12-mo-old male mice following 48 h fasting, related to **Figure 7F**.

Mean intensity (A.U.)	Control	TSC1mKO	4EBP1mt-TSC1mKO
<20	0	72.9 ± 24.3***	13.6 ± 22.1$^{\#\#}$
20-130	18.1 ± 13.9	27.1 ± 24.3	31.1 ± 25.8
130-350	41.1 ± 10.9	0*	32.2 ± 14.7
>350	40.7 ± 7.57	0*	23.2 ± 40.1

Percentage of total fibers in each intensity class is shown as mean ± SD. N=3/genotype; data was analysed by two-way ANOVA with Tukey's multiple comparison test (across rows). *P<0.05 and ***P<0.001 indicate significance vs control mice. ##P<0.01 indicates significance vs TSC1mKO mice.

Table S10. Quantification of western blot analysis in QM muscle acid lysate from 12-mo-old male mice following 18 h fasting, related to **Figure 8B**.

	**Control**	**TSC1mKO**	**4EBP1mt-TSC1mKO**
LAMP1	1 ± 0.28	1.69 ± 0.23*	1.88 ± 0.12**
Cathepsin L^{total}	1 ± 0.15	1.90 ± 0.36**	2.60 ± 0.29***$^{\#}$
Cathepsin $L^{immature}$	1 ± 0.24	1.29 ± 0.31	1.37 ± 0.37
Cathepsin L^{mature}	1 ± 0.38	3.30 ± 0.94*	5.41 ± 1.36**

Relative protein changes are shown as mean ± SD, vs control mice. N=3/genotype; each protein was analysed by one-way ANOVA with Tukey's multiple comparison test. *P<0.05, **P<0.01 and ***P<0.001 indicate significance vs control mice. #P<0.05; indicates significance vs TSC1mKO mice.

Table S11. Quantification of Lysotracker Red staining in QM from control male mice following 18 h fasting, related to **Figure 8E**.

Mean intensity (A.U.)	12 mo	26 mo
<15	0	54.2 ± 16.6***
15-40	8.82 ± 15.3	39.9 ± 19.1*
40-100	64.3 ± 8.42	5.19 ± 8.98****
>100	26.9 ± 9.04	0.74 ± 1.28

Percentage of total fibers in each intensity class is shown as mean ± SD. N=3/genotype; data was analysed by two-way ANOVA with Sidak's multiple comparison test (across rows). *P<0.05; ***P<0.001, ****P<0.0001 indicates significance between age in control mice.

Supplemental Reference

1. Levy Y, Ross, J. A., Niglas, M., Snetkov, V. A., Lynham, S., Liao, C. Y., Puckelwartz, M. J., Hsu, Y. M., McNally, E. M., Alsheimer, M., Harridge, S. D., Young, S. G., Fong, L. G., Español, Y., Lopez-Otin, C., Kennedy, B. K., Lowe, D. A., Ochala, J. Prelamin A causes aberrant myonuclear arrangement and results in muscle fiber weakness. JCI Insight. 2018;3:e120920.

2. Tsai S, Sitzmann, J.M., Dastidar, S.G., Rodriguez, A.A., Vu, S.L., McDonald, C.E., Academia, E.C., O'Leary, M.N., Ashe, T.D., La Spada, A.R., Kennedy, B.K. Muscle-specific 4E-BP1 signaling activation improves metabolic parameters during aging and obesity. J Clin Invest. 2015;125:2952-64.

3. Yamada E, Bastie, C. C., Koga, H., Wang, Y., Cuervo, A. M., Pessin, J. E. Mouse skeletal muscle fiber-type-specific macroautophagy and muscle wasting are regulated by a Fyn/STAT3/Vps34 signaling pathway. Cell Rep. 2012;1:557-69.

4. Steffen KK, MacKay VL, Kerr EO, Tsuchiya M, Hu D, Fox LA, et al. Yeast life span extension by depletion of 60s ribosomal subunits is mediated by Gcn4. Cell. 2008;133:292-302.

5. Williamson DL, Kubica N, Kimball SR, Jefferson LS. Exercise-induced alterations in extracellular signal-regulated kinase 1/2 and mammalian target of rapamycin (mTOR) signalling to regulatory mechanisms of mRNA translation in mouse muscle. J Physiol. 2006;573:497-510.

6. Zomzely CE, Roberts S, Gruber CP, Brown DM. Cerebral protein synthesis. II. Instability of cerebral messenger ribonucleic acid-ribosome complexes. J Biol Chem. 1968;243:5396-409.

7. Dobin A, Davis, C. A., Schlesinger, F., Drenkow, J., Zaleski, C., Jha, S., Batut, P., Chaisson, M., Gingeras, T. R. STAR: ultrafast universal RNA-seq aligner. Bioinformatics. 2013;29:15-21.

8. Liao Y, Smyth, G. K., Shi, W. The Subread aligner: fast, accurate and scalable read mapping by seed-and-vote. Nucleic Acids Res. 2013;41:e108.

9. Love MI, Huber, W., Anders, S. Moderated estimation of fold change and dispersion for RNA-seq data with DESeq2. Genome Biol. 2014;15:550.

10. Huang D, Sherman, B. T., Lempicki, R. A. Systematic and integrative analysis of large gene lists using DAVID bioinformatics resources. Nat Protoc. 2009;4:44-57.

11. Zhou Y, Zhou, B., Pache, L., Chang, M., Khodabakhshi, A. H., Tanaseichuk, O., Benner, C., Chanda, S. K. Metascape provides a biologist-oriented resource for the analysis of systems-level datasets. Nat Commun. 2019;10:1523.

12. Encarnacion-Rivera L, Foltz, S., Hartzell, H. C., Choo, H. Myosoft: An automated muscle histology analysis tool using machine learning algorithm utilizing FIJI/ImageJ software. PLoS One. 2020;15:e0229041.

13. Brüning JC, Michael, M. D., Winnay, J. N., Hayashi, T., Hörsch, D., Accili, D., Goodyear, L. J., Kahn, C. R. A muscle-specific insulin receptor knockout exhibits features of the metabolic syndrome of NIDDM without altering glucose tolerance. Molecular Cell. 1998;2:559-69.

14. Laboratory TJ. B6.FVB(129S4)-Tg(Ckmm-cre)5Khn/J. 2021. https://www.jax.org/strain/006475.

15. Kwiatkowski DJ, Zhang, H., Bandura, J. L., Heiberger, K. M., Glogauer, M., el-Hashemite, N., Onda, H. A mouse model of TSC1 reveals sex-dependent lethality from liver hemangiomas, and up-regulation of p70S6 kinase activity in Tsc1 null cells. Hum Mol Genet. 2002;11:525-34.

16. Smith MA, Katsouri, L., Irvine, E. E., Hankir, M. K., Pedroni, S. M., Voshol, P. J., Gordon, M. W., Choudhury, A. I., Woods, A., Vidal-Puig, A., Carling, D., Withers, D. J. Ribosomal S6K1 in POMC and AgRP Neurons Regulates Glucose Homeostasis but Not Feeding Behavior in Mice. Cell Rep. 2015;11:335-43.

Supplemental Figures

Title: Activation of eIF4E-binding-protein-1 rescues mTORC1-induced sarcopenia by expanding lysosomal degradation capacity.

Authors and affiliations:

Elisa M. Crombie[1], Seonyoung Kim[1], Stuart Adamson[3], Han Dong[1], Tzu-Chiao Lu[9], Yiju Wu[1], Yajun Wu[4], Yotam Levy [5], Nolan Stimple[3], Wing Moon R. Lam[6], Hwee Weng D. Hey[6], Dominic J. Withers[7], Ao-Lin Hsu[9, 10], Boon Huat Bay[4], Julien Ochala[5,8], Shih-Yin Tsai[1,2]

Supplemental Figures and legends

Supplemental Figure S1-S7

Supplementary Figures

Figure S1. Representative confocal images of immunofluorescence staining on negative control were taken from TSC1mKO mouse quadriceps muscle.

(**A**) DHE staining (left) compared to the negative control where only PBS was added (right). Scale bar, 20 μm. (**B**) Immunofluorescence staining (top panel) compared to the negative control where only secondary Ab and DAPI was added (bottom panel).

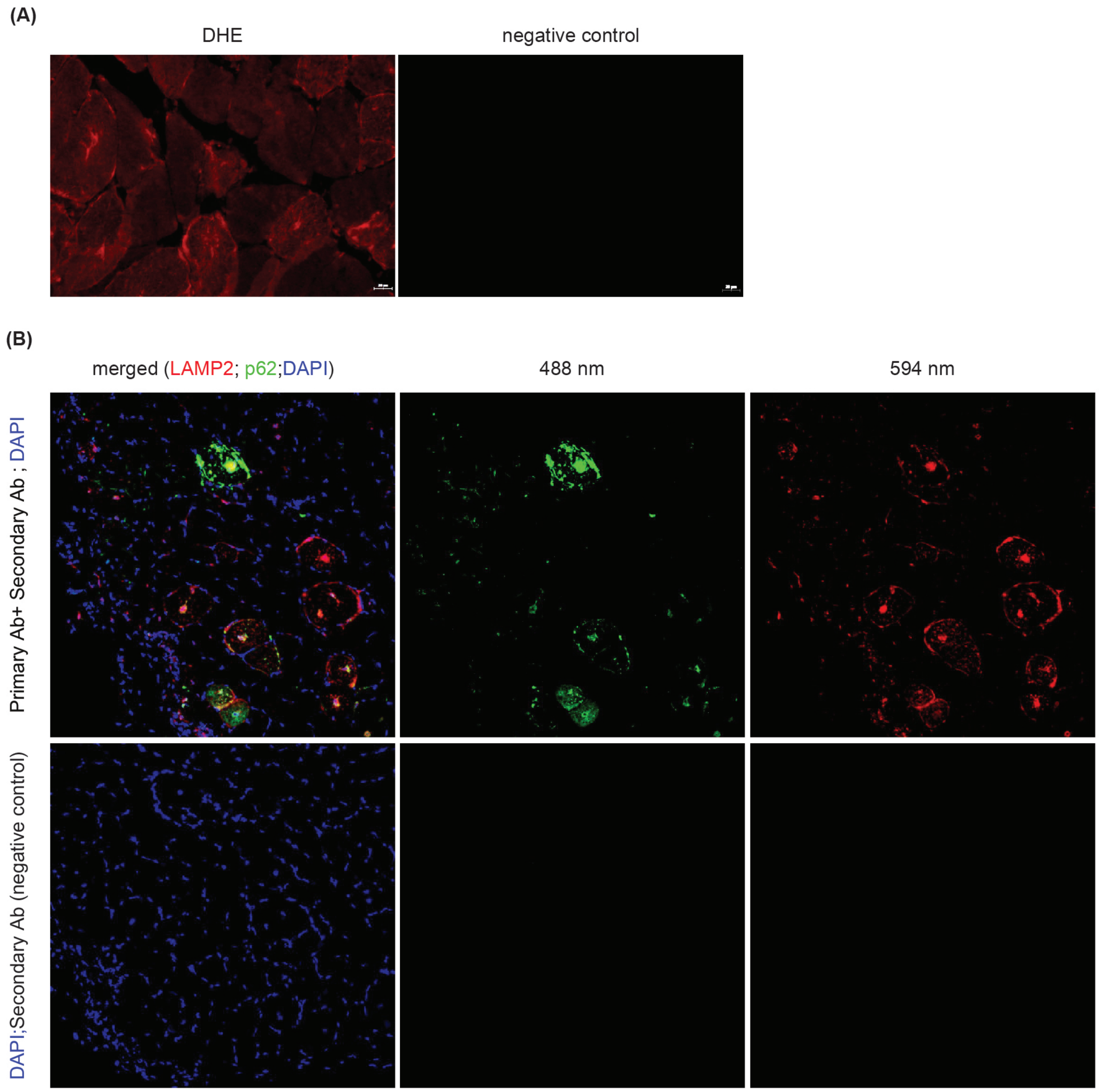

(A)
DHE
negative control
(B)
merged (LAMP2; p62;DAPI)
488 nm
594 nm
Primary Ab+ Secondary Ab ; DAPI
DAPI;Secondary Ab (negative control)

Figure S1

Figure S2. Molecular and physiological characterization of TSC1mKO, S6K1-TSC1mKO and 4EBP1mt-TSC1mKO mice.

(A) Myofiber specific force measured using individual myofibers ex vivo, normalised by myofiber CSA. 12-mo-old male mice were used for analyses. **(B)** Western blot analysis of mTORC1 canonical signalling from 12-mo-old male mouse QM samples. Ponceau S was used as the loading control. The quantification of **Figure S2B** is in **Table S4. (C)** Western blot analysis of mTORC1 canonical signalling from 4-mo-old male mouse QM samples. **(D)** Ponceau S membrane staining of **Figure 1C**. (**E**) The blot with additional samples accompanying for **Figure 1C**. (**F**) The accompanying blots for **Figure 1E** and **1F** of m7GTP pull-down assay of muscle lysates from 4-mo-old male mice. Brief**:** C, Control; T, TSC1mKO; ST, S6K1-TSC1mKO; ET, 4EBP1mt-TSC1mKO. (**G**) Computerized tomography (CT) scans showing spines of 12-mo-old male mice; representative images shown. (**H**) Kyphosis shown by Cobb angle measured from CT scan images from 12-mo-old male mice (Control; n=6, TSC1mKO; n=7, 4EBP1mt-TSC1mKO; and n=2, S6K1-TSC1mKO).

Data are shown as mean ± SEM and individual points correspond to one mouse. Statistical significance was determined by one-way ANOVA with Tukey's multiple comparison test. Only $P<0.05$ is labelled. *$P<0.05$, **$P<0.01$, ****$P<0.0001$ indicates statistical significance.

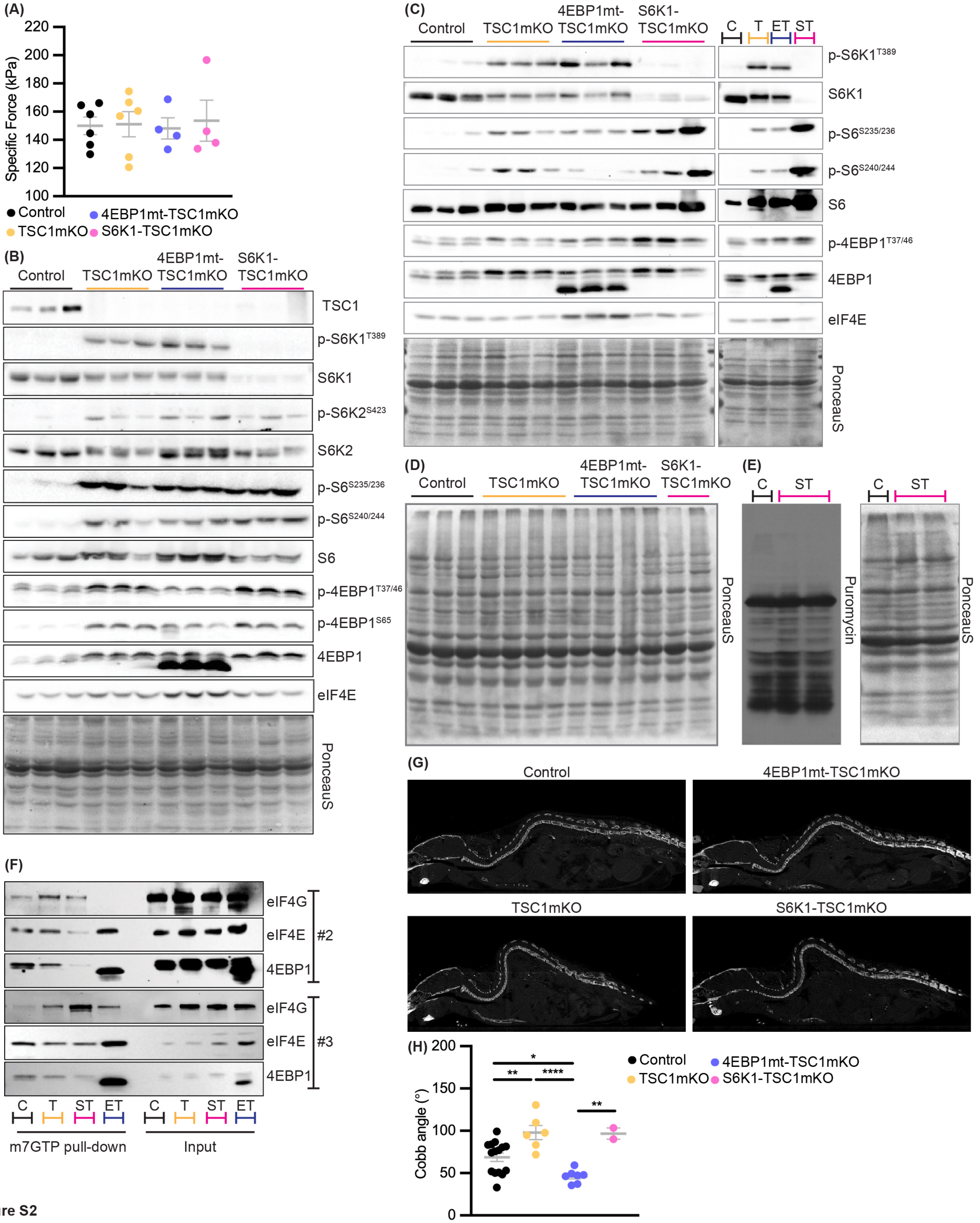
(A)
Specific Force (kPa)
Control
TSC1mKO
4EBP1mt-TSC1mKO
S6K1-TSC1mKO
(B)
Control
TSC1mKO
4EBP1mt-TSC1mKO
S6K1-TSC1mKO
TSC1
p-S6K1T389
S6K1
p-S6K2S423
S6K2
p-S6S235/236
p-S6S240/244
S6
p-4EBP1T37/46
p-4EBP1S65
4EBP1
eIF4E
PonceauS
(C)
C
T
ET
ST
(D)
(E)
Puromycin
(F)
eIF4G
#2
#3
m7GTP pull-down
Input
(G)
(H)
Cobb angle (°)

Figure S2

Figure S3. 4EBP1 activation ameliorated sarcopenic pathology in TSC1mKO mouse muscle.

Quantification of immunofluorescence staining of (**A**) type IIb, (**B**) type IIx, (**C**) type IIa, and **(D)** type I in cross-sections of whole GM from 12-mo-old male mice (Control n=3, TSC1mKO n=3, 4EBP1mt-TSC1mKO n=2, S6K1-TSC1mKO n=3). Myofiber size is quantified by minimal Feret diameter, with ~6000-11000 myofibers analysed per mouse. Data are shown as mean ± SEM and statistical significance was determined by two-way ANOVA with Tukey's multiple comparison test compared within each size class. *P<0.05, **P<0.01, ***P<0.001, ****P<0.0001 indicates statistical significance of TSC1mKO vs control mice; $^{\#}$P<0.05, $^{\#\#}$P<0.01, $^{\#\#\#}$P<0.001, $^{\#\#\#\#}$P<0.0001 indicates significance of 4EBP1mt-TSC1mKO (blue) or S6K1-TSC1mKO (pink) vs TSC1mKO mice. **(E)** H&E staining of QM from 12-mo-old male mice. Black arrows, myofibers with inclusions; white arrows, degenerated basophilic fibers; * basophilic "ragged" fibers. Scale bar, 50 µm. (**F**) Co-localisation analysis of multiple stains on serial sections of 12-mo-old male mouse QM; scale bar, 100 µm. MyHC staining was analysed by either combination of type IIa and type I with either type IIb (first column) or type IIx (second column). SDH staining is used for assessment of mitochondrial activity (third column). Corresponding muscle types are labelled in SDH image for easy reference. Yellow font indicates abnormal type IIb myofibers with intense SDH staining. (**G**) RT-PCR for mitochondrial genes, using RNA extracted from 12-mo-old male mouse GM. Data are shown as mean ± SEM. Statistical significance was determined by one-way ANOVA across rows with Tukey's multiple comparison test. Only P<0.05 were labelled. **(H)** Ponceau S membrane staining of **Figure 3A**. (**I**) The blot with additional samples accompanying for **Figure 3A** of OXPHOS proteins from 12-mo-old male mouse QM, where each representative protein from each mitochondrial complex is

indicated, and the quantification is shown in **Table S4**. Brief**:** C, Control; T, TSC1mKO; ST, S6K1-TSC1mKO; ET, 4EBP1mt-TSC1mKO.

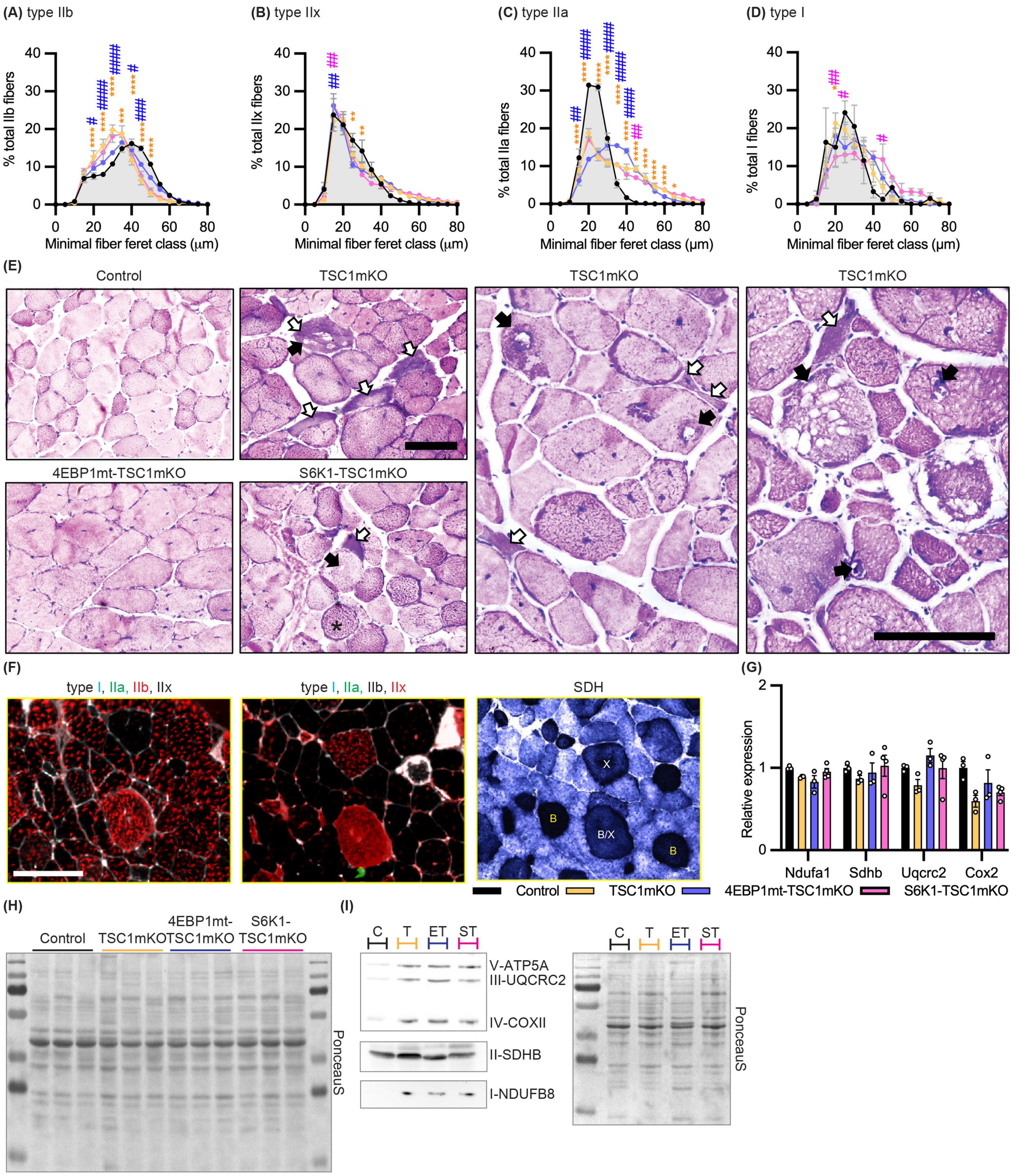

(A) type IIb
(B) type IIx
(C) type IIa
(D) type I
% total IIb fibers
% total IIx fibers
% total IIa fibers
% total I fibers
Minimal fiber feret class (µm)
(E)
Control
TSC1mKO
4EBP1mt-TSC1mKO
S6K1-TSC1mKO
(F)
type I, IIa, IIb, IIx
type I, IIa, IIb, IIx
SDH
X
B
B/X
B
(G)
Relative expression
Ndufa1
Sdhb
Uqcrc2
Cox2
Control
TSC1mKO
4EBP1mt-TSC1mKO
S6K1-TSC1mKO
(H)
Control
TSC1mKO
4EBP1mt-TSC1mKO
S6K1-TSC1mKO
PonceauS
(I)
C
T
ET
ST
V-ATP5A
III-UQCRC2
IV-COXII
II-SDHB
I-NDUFB8
C
T
ET
ST
PonceauS

Figure S3

Figure S4. RNA-seq analysis of transcriptome and translatome from 2-mo-old TSC1mKO male mouse skeletal muscle.

Representative polysome profiles of limb muscles from 2-mo-old male (**A**) Control, (**B**) TSC1mKO, and (**C**) S6K1-TSC1mKO mice. Polysome profile image (top), RNA gel labelled with ribosomal RNA loaded from each fraction (middle) and immunoblot (bottom) of cytoplasmic marker HSP90 and ribosomal marker RPL24. The selected polysome fractions for RNA-seq analysis are indicated. (**D**) Venn diagrams indicating number of differentially expressed genes common to the transcriptome or translatome from RNA-seq of total and polysome RNA, respectively. TSC1mKO vs control mice; direction of regulation is indicated. (**E**) Enriched Gene Ontology (GO) terms by Metascape analysis of differentially expressed genes in the translatome.

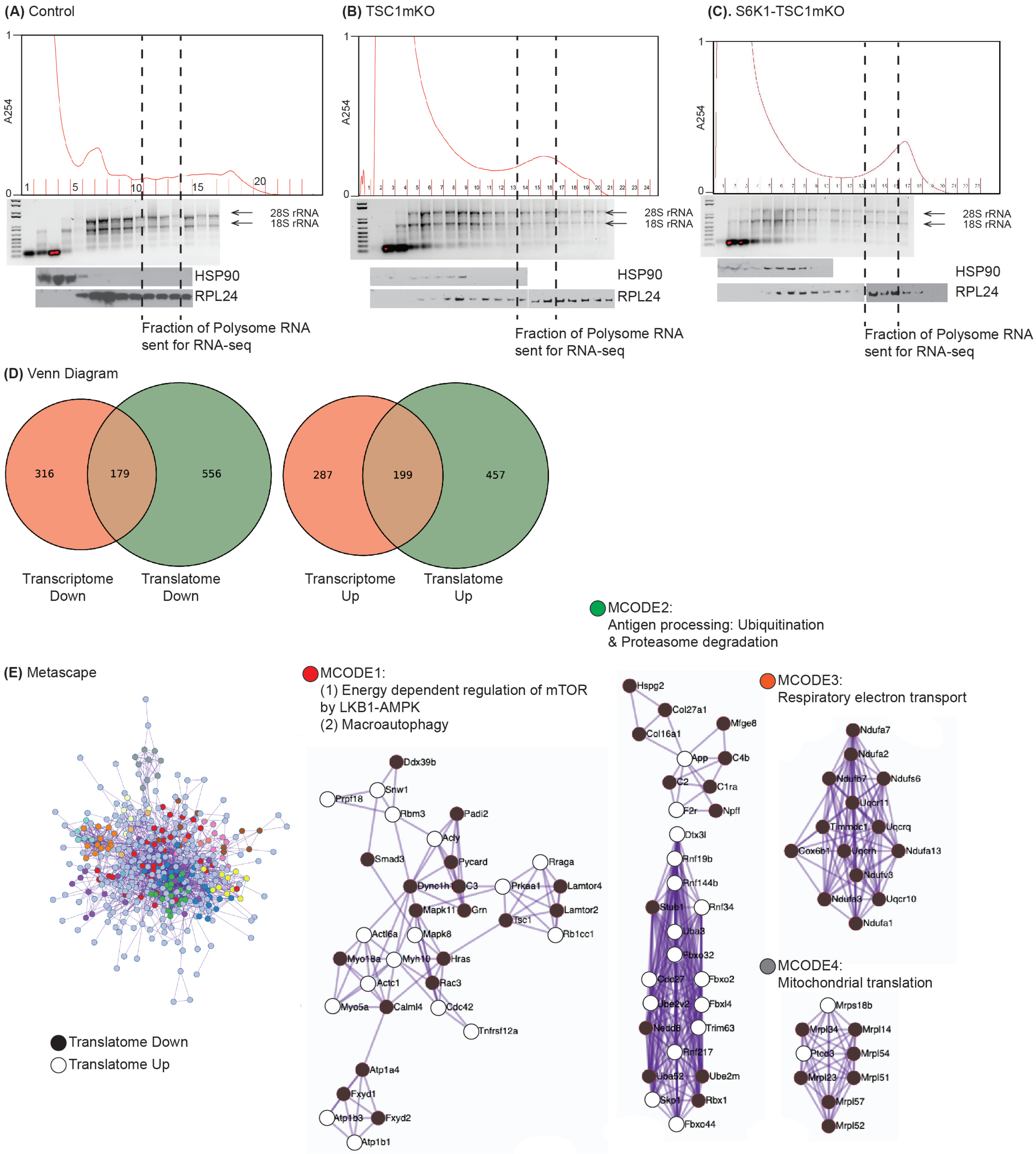

(A) Control
(B) TSC1mKO
(C). S6K1-TSC1mKO
A254
28S rRNA
18S rRNA
HSP90
RPL24
Fraction of Polysome RNA sent for RNA-seq
(D) Venn Diagram
316
179
556
287
199
457
Transcriptome Down
Translatome Down
Transcriptome Up
Translatome Up
(E) Metascape
Translatome Down
Translatome Up
MCODE1:
(1) Energy dependent regulation of mTOR by LKB1-AMPK
(2) Macroautophagy
MCODE2:
Antigen processing: Ubiquitination & Proteasome degradation
MCODE3:
Respiratory electron transport
MCODE4:
Mitochondrial translation
Ddx39b
Snw1
Prpf18
Rbm3
Padi2
Acly
Smad3
Pycard
Rraga
Dync1h1
C3
Prkaa1
Lamtor4
Mapk11
Grn
Tsc1
Lamtor2
Actl6a
Mapk8
Rb1cc1
Myo18a
Myh10
Hras
Actc1
Rac3
Myo5a
Calml4
Cdc42
Tnfrsf12a
Atp1a4
Fxyd1
Atp1b3
Fxyd2
Atp1b1
Hspg2
Col27a1
Mfge8
Col16a1
App
C4b
C2
C1ra
F2r
Npff
Dtx3l
Rnf19b
Rnf144b
Stub1
Rnf34
Uba3
Fbxo32
Cdc27
Fbxo2
Ube2v2
Fbxl4
Nedd8
Trim63
Rnf217
Uba52
Ube2m
Skp1
Rbx1
Fbxo44
Ndufa7
Ndufa2
Ndufb7
Ndufs6
Uqcr11
Timmdc1
Uqcrq
Cox6b1
Uqcrh
Ndufa13
Ndufv3
Ndufa3
Uqcr10
Ndufa1
Mrps18b
Mrpl34
Mrpl14
Ptcd3
Mrpl54
Mrpl23
Mrpl51
Mrpl57
Mrpl52

Figure S4

Figure S5. The loading control and accompany immunoblotting images for Figure 5.

(**A**) Ponceau S membrane staining of **Figure 5A.** (**B**) The blot with additional samples accompanying for **Figure 5A**. Immunoblotting of proteins in insoluble fraction of protein lysate from 12-mo-old male mouse QM; Ponceau S membrane staining was used as a loading control. The detailed quantification of **Figure 5A** and **S5B** is presented in **Table S5.** Brief**:** C, Control; T, TSC1mKO; ST, S6K1-TSC1mKO; ET, 4EBP1mt-TSC1mKO. (**C**) Immunoblotting of autophagy markers from 12-mo-old male mouse QM; Ponceau S membrane staining was used as a loading control. The quantification is shown in **Table S4**. (**D**) Ponceau S membrane staining of **Figure 5D.** (**E**) Immunoblotting of muscle incubated in the presence or absence of NH_4Cl and leupeptin (Leu) to inhibit lysosomal degradation. This assay is shown as three independent blots for **Figure 5C** quantification. The quantification is shown in **Table S7.** (**F)** Immunoblotting of autophagy markers during prolonged fasting intervals in 12-mo-old male mouse QM and Ponceau S membrane staining was used as a loading control. This assay is shown as three independent replicates for **Figure 5E**.

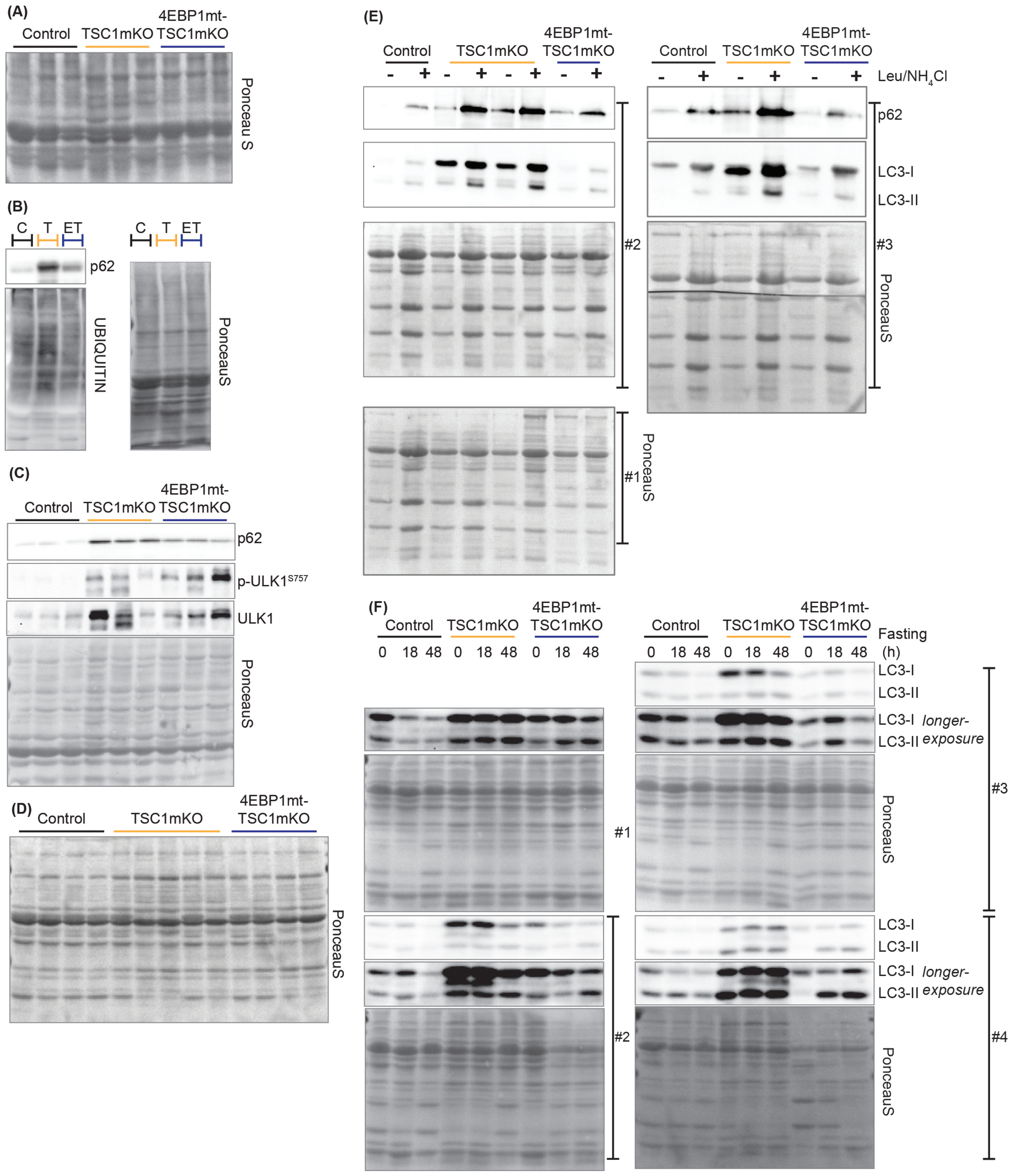
(A)
Control
TSC1mKO
4EBP1mt-
TSC1mKO
Ponceau S
(B)
C
T
ET
p62
UBIQUITIN
PonceauS
(C)
p-ULK1S757
ULK1
(D)
(E)
Leu/NH4Cl
LC3-I
LC3-II
#1
#2
#3
(F)
Fasting
(h)
0
18
48
longer-
exposure
#4

Figure S5

Figure S6. Activation of 4EBP1 lowered proteostatic stress in TSC1mKO mouse muscle.

(**A**) Immunofluorescence staining on cross-sections of 12-mo-old male GM. DAPI staining was used to label nuclei and laminin was used to outline the myofibers. (**B**) Immunofluorescent staining for p62 (green), LAMP1 (red), and Laminin (white) and (**C**) immunofluorescent staining for MyHC and Laminin (white) in whole GM from 12-mo-old male mice (images by TissueFAXS). Yellow signal indicates co-localisation of p62 and LAMP1. Scale bar, 500 μm.

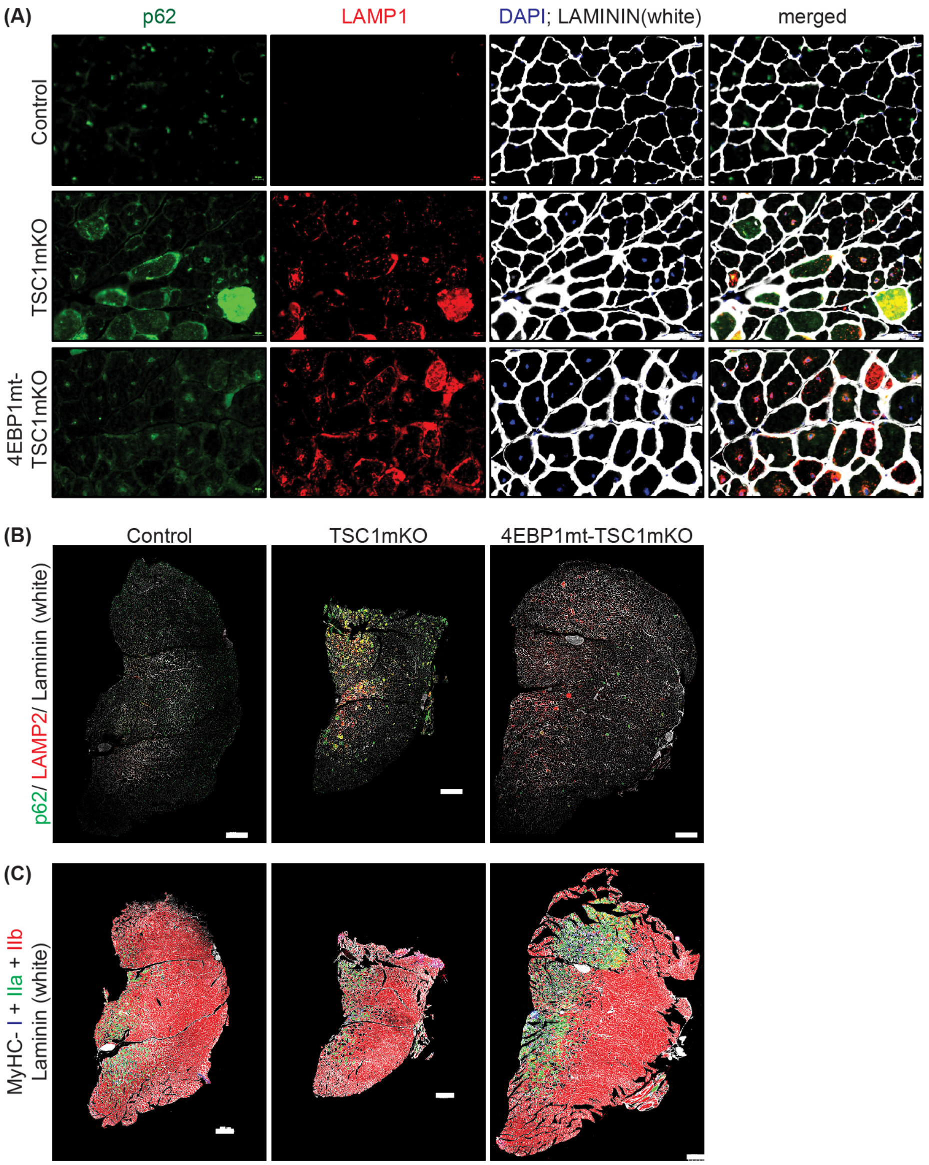

(A)
p62
LAMP1
DAPI; LAMININ(white)
merged
Control
TSC1mKO
4EBP1mt-TSC1mKO
(B)
Control
TSC1mKO
4EBP1mt-TSC1mKO
p62/ LAMP2/ Laminin (white)
(C)
MyHC- I + IIa + IIb
Laminin (white)

Figure S6

Figure S7. Lysosomal pH is elevated in TSC1mKO mouse muscle.

(**A**) Ponceau S membrane staining of **Figure 7A.** (**B**) The blot with additional samples accompanying for **Figure 7A** with quantification in **Figure 7B** and **Table S6**. (**C**) Coomassie blue staining of **Figure 8B.** (**D**) LysoSensor Green staining of GM of 12-mo-old male mice fasted for 48 h. Scale bar, 50 μm.

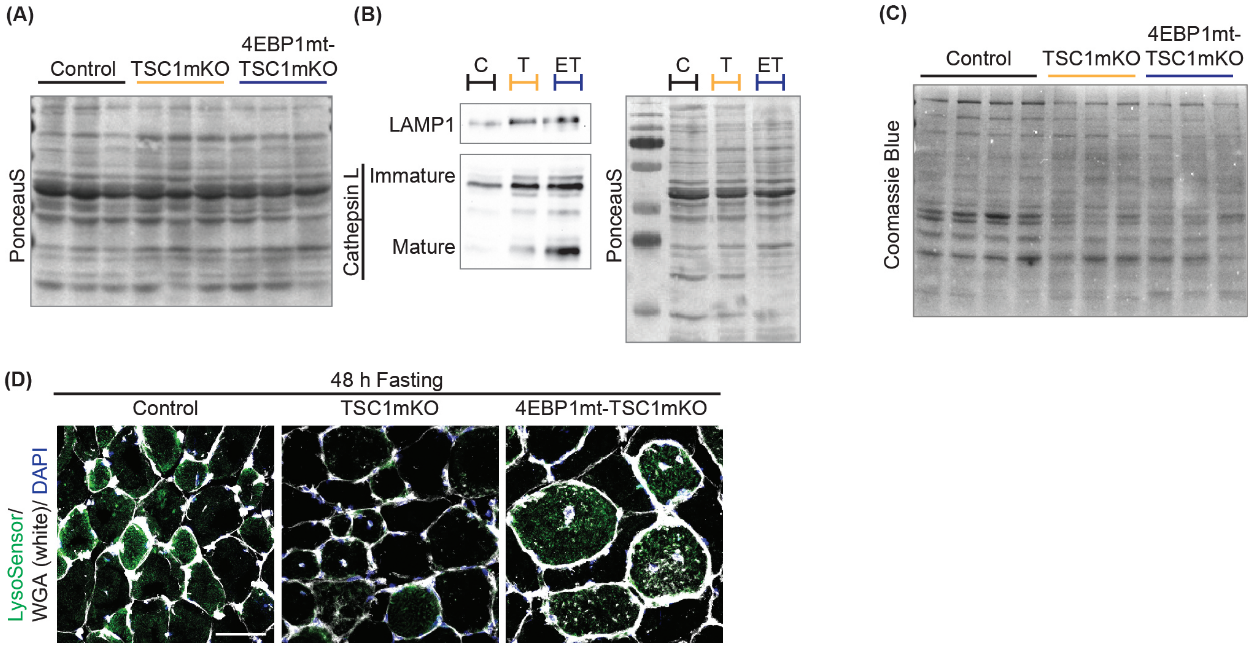
(A)
Control
TSC1mKO
4EBP1mt-TSC1mKO
PonceauS
(B)
C
T
ET
LAMP1
Cathepsin L
Immature
Mature
C
T
ET
PonceauS
(C)
Control
TSC1mKO
4EBP1mt-TSC1mKO
Coomassie Blue
(D)
48 h Fasting
Control
TSC1mKO
4EBP1mt-TSC1mKO
LysoSensor/ WGA (white)/ DAPI

Figure S7
